# The design and development of short peptide-based novel smart materials to prevent fouling by the formation of non-toxic and biocompatible coatings†

**DOI:** 10.1039/c9ra10018k

**Published:** 2020-04-01

**Authors:** Amutha Arul, Subramaniyam Sivagnanam, Ananta Dey, Oindrilla Mukherjee, Soumyajit Ghosh, Priyadip Das

**Affiliations:** Department of Chemistry, SRMIST SRM Nagar, Potheri, Kattankulathur, Kancheepuram District Chennai Tamil Nadu 603203 India priyadipcsmcri@gmail.com soumyajitghosh89@gmail.com; Academy of Scientific and Innovative Research (AcSIR) Ghaziabad – 201002 India; CSIR-Central Salt & Marine Chemicals Research Institute Bhavnagar 364002 India; Department of Biotechnology, National Institute of Technology Durgapur West Bengal – 713209 India

## Abstract

Biofouling refers to the undesirable process that leads to the accumulation of microorganisms such as bacteria or fungi on substrates. This is one of the major concerns associated with several components of our regular life such as food, health, water and energy. In the healthcare sector, biofouling on medical devices is known to cause infections, which are often resistant to conventional antibiotics and lead to increase in the number of hospital and surgery-related deaths. One of the better ways to tackle the problem of biofouling is the development of smart antifouling materials that can produce a biocompatible, non-toxic, eco-friendly and functional coating and maintain a biological environment without any adverse effect. To this end, in the present study, we have reported the design and synthesis of two simple chemically modified peptides, namely, PA1 (PFB-VVD) and PA2 (PFB-LLE). The design as well as the amino acid sequence of the peptides contains three basic components that enable their ability to (i) self-assemble into functional coatings, (ii) bind with the desired surface *via* the bi-dentate coordination of dicarboxylate groups and (iii) exhibit antifouling activity and generate a non-toxic biocompatible supramolecular coating on the desired surface. PA1 having aspartic acid as the anchoring moiety exhibits better antifouling activity compared to PA2 that has glutamic acid as the anchoring moiety. This is probably due to the greater adhesive force or binding affinity of aspartic acid to the examined surface compared to that of glutamic acid, as confirmed by force measurement studies using AFM. Most importantly, the simple drop-coating method promises great advantages due to its ease of operation, which leads to a reduction in the production cost and increase in the scope of commercialization. To the best of our knowledge, this is the first attempt to develop an ultra-short peptide-based smart antifouling material with a dicarboxylate group as the surface binding moiety. Furthermore, these findings promise to provide further insights into antifouling mechanisms in the future by the development of a smart material using a dicarboxylate group as an anchoring moiety.

## Abstract 2

Two newly synthesized peptides, PA1 and PA2 with fluorinated aromatic unit and dicarboxylate group as an anchoring moiety able to form a non-toxic, stable, coating by a simple drop-coating method, improving the antifouling property of the surface.

## Graphical abstract

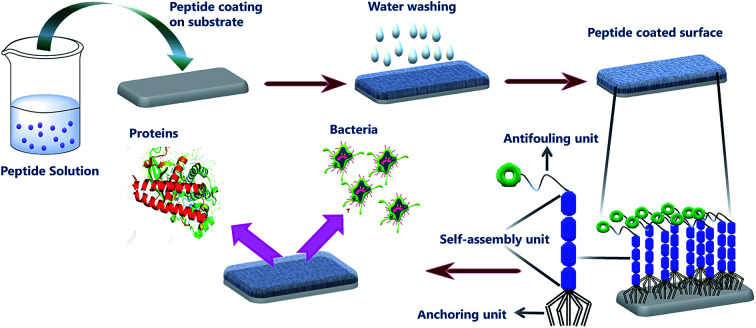

## Introduction

Biofouling is an adverse process in which several organisms such as bacteria, fungi, barnacles, bryozoans, and sponges encrust a surface. This process is initiated by the adsorption of several biomolecules, such as polysaccharides and proteins, followed by the accumulation of other organisms from the neighboring areas, finally leading to the formation of an ordered network on the surface.^1^ In hospitals, these organisms may consist of pathogenic bacteria that form ordered aggregates of bacterial colonies on the surface, which are then termed biofilms.^2^ Biofilm formation is a serious concern in many areas connected to our regular life such as healthcare systems,^3^ food packaging,^4^ water treatment,^5^ and marine industries.^6^ In particular, biofouling is an adverse event in healthcare, which can damage the functioning of medical expedients. Several examples of problematic biofouling include protein accumulation onto biosensor surfaces,^7^ the blocking of cardiovascular implants by thrombi,^8^ and the bacterial colonization of contact lenses and indwelling catheters.^9^ In many cases, it has been observed that patient infections and other complications from biofouling significantly increase the cost of healthcare delivery and can lead to compromised implant performance or even implant failure. Furthermore, the biofilm formation leads to the accumulation of bacteria and become resistant to usual antibiotics, which consequently poses a high risk on their utility.^10^ Therefore, considering biofouling to be a widespread phenomenon with high negative impacts on many areas related to our regular life, the development of cost-effective smart materials to resist fouling is in high demand.

Different strategies have been developed to resist biofouling based on chemical, physical, and topographical modifications of the desired surfaces.^11^ Considering the advantages and disadvantages of the various strategies, researchers have found that the use of coatings is the most promising approach to resist the adhesion of biomolecules, bacteria and other organisms onto surfaces. The materials used for this purpose are referred to as antifouling materials.^12^ Antifouling materials can serve as coatings on various surfaces to resist the bacterial adhesion as well as biofilm formation. Several research groups have developed various antifouling coatings to resist biofouling on the surface of biomedical implants. However, many of these have certain drawbacks, which include a lack of long-term stability, low biocompatibility and substantial toxicity. Therefore, there is an immense demand for the development of long-term stable, non-toxic and biocompatible antifouling coatings that sustain the biological environment without any adverse effect. This can be accomplished by designing smart antifouling materials from biomolecules or combining artificial active moieties with biomolecules or biocompatible molecules.^11*a*^ In this context, peptides as a building block have received much attention in an effort to develop smart antifouling materials due to their biocompatible, non-toxic and eco-friendly nature.^13^ In recent years, different types of peptides, such as self-assembled,^14^ PEGylated,^15^ polymer-grafted,^16^ zwitterionic,^17^ amphiphilic,^17*c*,18^ hydrogelators,^19^ and peptidomimetics,^20^ have been employed to develop antifouling coatings. Among the above-mentioned types of peptides, antifouling materials developed from self-assembled short peptides are of prime significance due to their ease of synthesis with tunable structural characteristics and low toxic profile. Furthermore, the short peptide-based self-assembled structural coatings also address the major concern, the stability of the coating. For the construction of this type of smart antifouling material, the design should contain three basic components: (i) a self-assembling unit to generate the supramolecular coating (ii) an anchoring unit to adsorb onto different substrates and (iii) an antifouling unit.

There are several reports on the development of antibacterial peptides; however, only a few of them exhibit antifouling activity. Most importantly, many of them comprise the unusual amino acid 3,4-dihydroxy phenylalanine (DOPA) with a catechol group as an anchoring moiety.^14*a*,*b*,16*a*,*d*,20*a*,*d*,21^ This choice is preferred because DOPA is the key constituent of adhesive proteins of marine mussels (mussel foot proteins (mfps))^22^ and is able to adhere to different surfaces.^14*a*,22*b*,*c*,*d*^ The oxidized form of DOPA plays an important role as a cross-linker agent that leads to the solidification of the secreted liquid protein adhesive.^23^ This adaptive binding of DOPA to different surfaces was further established by single molecule force spectroscopy using atomic force microscopy.^22*a*,*b*,24^ Recently, Reches and co-workers developed a tripeptide that self-assembles into a functional coating with antifouling activity. This tripeptide contains three units: (i) adhesive (ii) self-assembling and (iii) antifouling. They also chose 3,4-dihydroxyphenylalanine (DOPA) as the adhesive moiety for the attachment of the peptide with the chosen surface and the self-assembly unit included fluorinated phenylalanine residues.^14*a*^ In most of the cases, the synthetic procedures of the peptides involving DOPA have synthetic complications and need additional steps to avoid these difficulties. All of these make the synthetic procedure less cost-effective with a low yield of the desired products. On the other hand, the development of a multilayered antifouling coating involving an antifouling polymer with a catechol group as the surface anchor necessitates multistep treatments, which normally leads to an increase in the production cost. Therefore, the search still continues for other alternative anchoring moieties that are able to bind the synthesized peptides on the desired surface. Recently, Zhang and his group outlined a detailed mechanism to control the strength of gold–thiol interactions.^25^ Their experimental results revealed that an oxidized gold surface is able to increase the gold–thiol interaction and the binding modes of the interaction changes depending on the interaction time and pH of the environment.^25^ This result led to the design of coated surfaces based on gold–thiol interactions for a variety of bio-analytical applications. He and co-workers compared the antifouling efficiency between zwitterionic and amphiphilic peptide-based SAMs.^17*c*^ Amphiphilic peptides composed of alternating sequences of hydrophobic tyrosine (Y) and hydrophilic serine (S) residues (CYSYSYS) was compared with a zwitterionic peptide having alternating positively charged arginine residues (R) and negatively charged glutamic acid (E) residues (CRERERE). Both the peptides accumulated as self-assembled monolayers (SAMs) on gold substrates through a cysteine residue. However, the ultra-low fouling natural peptides, comprising negatively and positively charged amino acid residues in the form of either alternating or randomly mixed charge,^26^ have longer amino acid sequences and require an alkanethiol for the adhesion to gold.^27^ There are well-known basic studies about the interactions of a single amino acid with well-defined metal and metal oxide surfaces. It has been found that peptides can strongly bind with metal oxide surfaces such as titania or silica through the carboxylate moiety as a preferential binding site due to their strong electrostatic interactions with the charged surface.^28^ Burgi *et al.* described a protonation–deprotonation two-stage process accompanied by the reorientation of molecules, in which a rapid deprotonation of COOH from glutamic acid is followed by a slow deprotonation of the COOH group from the glycine residue assisted by the adsorption of a carboxylate group on the Au surface.^29^ Zwitterionic peptides interact with a TiO_2_ surface in a bidentate coordination fashion in which the carboxylate group is bound to two Ti atoms, which exhibits the preferential binding of the carboxylate group with the surface Ti cations. This explains the amino acid adsorption on a TiO_2_ surface.^30^ This fact can enable further investigations aimed at controlling the biocompatibility or biofilm growth process. With this motivation, herein we designed and synthesized two chemically modified short peptides PA1 (PFB-VVD) and PA2 (PFB-LLE) (V = Val, L = Leu, E = Glu, D = Asp, PFB = pentafluorobenzaldehyde) which comprise three basic elements: (i) self-assembly unit (ii) anchoring moiety and (iii) antifouling moiety. Both these peptides are expected to bind with the examined surface using the dicarboxylate group as an anchoring moiety and the pentafluoro-substituted benzene ring as the antifouling unit. We used a simple drop-coating method to coat the desired surfaces and increase their antifouling activity, avoiding the multistep sophisticated techniques used for surface pre-treatment as well as modification and reducing the production cost. These low molecular weight peptides can easily form a functional coating on a desired surface by a simple drop-casting technique and demonstrate the ability to interrupt the biofouling process. These newly synthesized peptide-based coatings exhibited their aptitude in terms of resolving two major problems common to implanted metal oxide-based surfaces: (i) non-specific protein adsorption and (ii) bacterial colonization. Substrates coated with PA1 exhibited better antifouling activity in comparison to PA2 due to the greater adhesive force or binding interaction of aspartic acid than glutamic acid as an anchoring moiety. To the best our knowledge, this is the first report on the development of ultra-short (low molecular weight) peptide-based smart antifouling materials with a dicarboxylate group as an anchoring moiety.

## Results and discussion

**Scheme 1:**
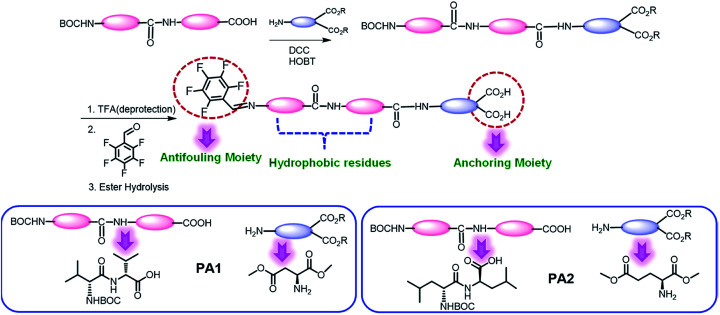
Schematic representation for the synthesis of the desired tripeptides PA1 and PA2.

We synthesized two peptides PA1 (PFB-VVD) and PA2 (PFB-LLE) with a Val–Val–aspartic acid and Leu–Leu–glutamic acid amino acid sequence, respectively. In both the peptides, the terminal amine groups are coupled with 2,3,4,5,6-pentafluorobenzaldehyde (PFB) through an imine bond (Scheme 1). All the synthesized products were isolated and characterized by standard analytical techniques (ESI†) (Scheme 1, details of the synthetic procedure are provided in the Experimental section, Schemes S1–S6, ESI†). The design we chose was one in which two adjacent hydrophobic amino acids (VV-in PA1 and LL in PA2) directed the self-assembly of these peptides owing to the hydrophobic interactions and formed highly ordered supramolecular structures. We can expect that this motif will direct the self-assembly of the peptides into a film or functional coating. The last amino acids of these peptides (PA1, PA2) are aspartic acid and glutamic acid that have a dicarboxylate functional group as an anchoring moiety, which help to bind the peptides with the desired surfaces. Lysine peptides (*n* = 2–5) and polylysine (*n* = 169) are reported to bind with TiO_2_ particle-based films from aqueous solutions and the carboxylate group was specifically involved in the peptide–TiO_2_ binding interaction.^30*c*^ Furthermore, we anticipated that the carbon–fluorine bond of the pentafluoro-substituted aromatic ring would lead to the construction of a “Teflon-like” material that will prevent the non-specific adsorption of proteins to the surface as well as bacterial colonization and therefore can play the role of an antifouling moiety.^14*a*^

**Fig. 1:**
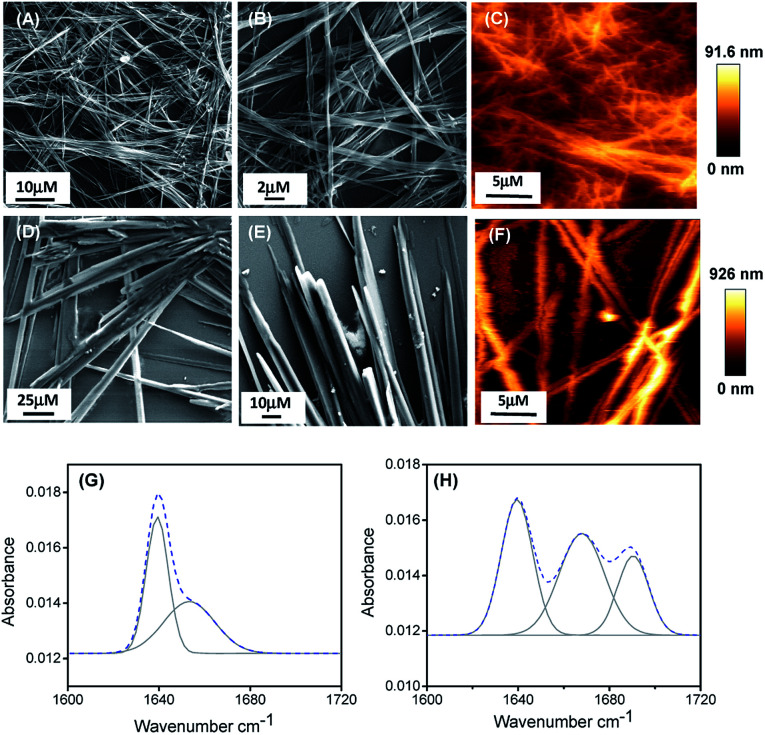
FE-SEM micrographs of the self-assembled structure formed by PA1 (A and B) and PA2 (D and E) in 50% aqueous ethanol. AFM micrographs (two dimensional representation) of the self-assembled structure formed by PA1 (C) and PA2 (F). Deconvoluted FT-IR spectra of the self-assembled structures formed by PA1 (G) and PA2 (H). The dashed line indicates the experimental FTIR spectrum and the solid line represents the deconvoluted curves with a Gaussian function.

We have studied the self-assembly property of both the newly synthesized chemically-modified oligopeptides (PA1 and PA2) in an aqueous ethanol medium. To trigger the self-assembly process, the peptides were dissolved in 1,1,1,3,3,3-hexafluoro-2-propanol (HFP). Then, we diluted each solution in 50% aqueous ethanol medium (1 : 1 EtOH/H_2_O) to get a final effective concentration of 3 mg mL^−1^. Field emission scanning electron microscopy (FE-SEM) analysis revealed that PA1 formed a fibrillar network consisting of thin fibril structures (Fig. 1A and B), while PA2 formed a tubular structure (Fig. 1D and E).

AFM analysis further supported the results obtained by SEM and showed a similar morphology of the self-assembled state for both the peptides (Fig. 1C and F). We also studied the self-assembly property of both these peptides PA1 and PA2 in 100% ethanol and water systems. PA1 self-assembled into a thin fibrillar network in ethanol but in water, could not self-assemble to give any definite architectures. On the other hand, PA2 self-assembled into an aggregated tubular structure in ethanol and a branched tubular structure in an aqueous medium (Fig. S15, ESI†).

**Fig. 2:**
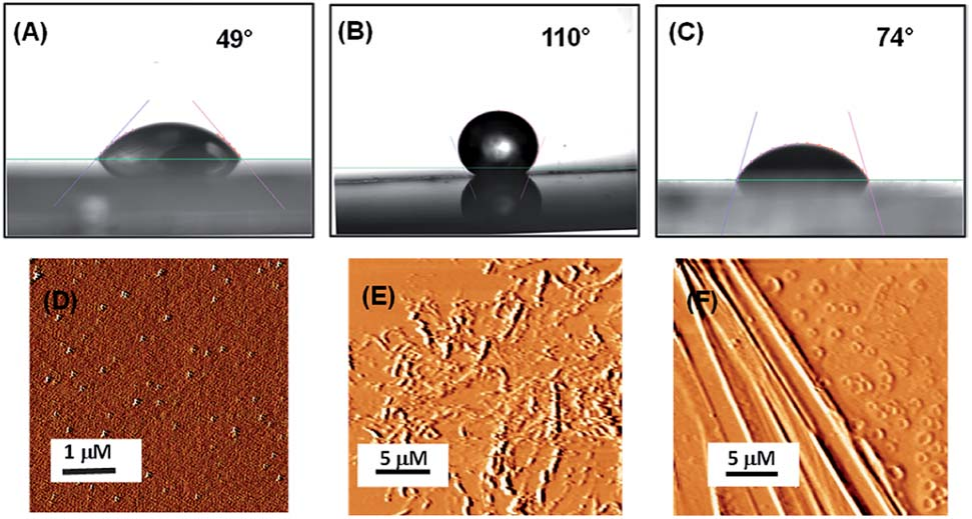
Surface characterization of bare silica surface and silica surface coated with peptides PA1 and PA2. Contact angle measurements of (A) uncoated silica, (B) coated with peptide PA1, (C) coated with peptide PA2. AFM topography images of (D) uncoated silica, (E) coated with peptide PA1, (F) coated with peptide PA2.

To elucidate the secondary conformation of the self-assembled supramolecular structures formed by PA1 and PA2, we used Fourier transform infrared (FTIR) spectroscopy and deconvoluted each spectrum in the amide-I region with a Gaussian function. The FT-IR spectrum of the thin fibrillar structure formed by PA1 exhibits one major peak at 1640 cm^−1^ and one minor peak at 1653 cm^−1^, ascribed to the considerable disorder or random structures (Fig. 2H).^31^ The tubular structure formed by PA2 exhibited three distinctive peaks at 1638 cm^−1^, 1667 cm^−1^ and 1691 cm^−1^ (Fig. 2G). The peaks at 1638 cm^−1^ and 1691 cm^−1^ suggest the presence of an anti-parallel β-sheet secondary conformation^31*a*,32^ and another peak at 1667 cm^−1^ corresponds to β-turn conformation.^31*a*,32*c*^ We obtained similar information on the secondary structure of the peptides using circular dichroism (CD) spectroscopy. The CD spectral pattern of PA1 did not contain the positive peak at ∼220–225 nm but possessed a red-shifted negative band at ∼230–240 nm (Fig. S16A, ESI†). This reflects an increase in the motional flexibility of PA1 and some attendant loss of the secondary structural conformation.^33^ On the other hand, the CD spectrum of PA2 showed a characteristic negative peak at ∼210 nm and a positive peak at ∼200 nm (Fig. S16B, ESI†). This spectral pattern indicates that PA2 has an anti-parallel β-sheet conformation.^34^

In order to evaluate the antifouling activity of these two peptides PA1 and PA2, we coated a silica (SiO_2_) surface with them. To obtain the peptide-coated surface, substrates were coated with the peptide assemblies (3 mg mL^−1^) by allowing the peptides to self-assemble and then drop-casting the peptide solution onto a clean silica surface. After the surfaces dried in air, they were carefully washed with distilled water to remove the remaining non-adhered peptides. Finally, the surfaces were dried under nitrogen. Due to the peptide adsorption on the surface, a Teflon-like layer was formed. We used ethanol as a solvent system for coating the surface since it dissolves the peptides completely while allowing them to adhere on the substrate.

Understanding the interaction of the peptide-coated surfaces with water is essential as both hydrophobicity and hydrophilicity play a significant role in the design of smart and efficient antifouling materials.^35^ It was observed that the hydrophobic nature of the peptide-coated surfaces was increased compare to that of the bare surface. The increased hydrophobicity of the Teflon-like layer coated surfaces was confirmed by contact angle measurements with water droplets. The results showed an increase in the water contact angle from 49° to 110° (with the PA1 coated surface) and from 49° to 74° (with the PA2 coated surface) (Fig. 2A–C). Furthermore, there is a considerable increase in the water contact angle of the coated surfaces with an increasing concentration of the peptide solution (Fig. S9, ESI†). This correlation between the angle size and peptide concentration is due to the presence of hydrophobic side chain amino acid residues in the peptide backbone. These results evidently revealed the formation of a more hydrophobic coating by peptide PA1 in comparison to PA2.

We characterized the topography of the uncoated silica surface (oxidized Si surface by thermal annealing) and silica surface coated with these peptides (PA1 and PA2) using atomic force microscopy (AFM). AFM analysis clearly revealed that there is a substantial difference in the topography of the peptide-coated silica surfaces and bare silica surface. In addition, some self-assembled supramolecular aggregates of the respective peptides appeared on the coated substrate (Fig. 2D–F).

**Fig. 3:**
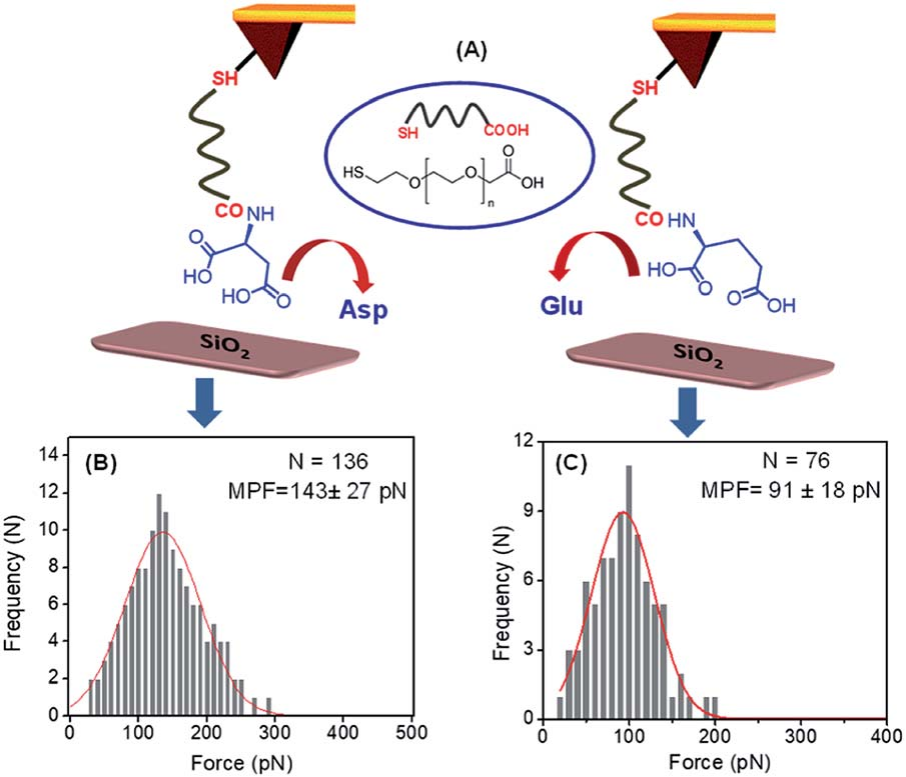
(A) Pictorial representation of the binding interaction between the Asp/Glu-functionalized AFM tip (Au) and the SiO_2_ surface (prepared by annealing the bare Si in the presence of O_2_ at higher temperatures). Histograms of the adhesive force for the binding interaction values for (B) aspartic acid, (C) glutamic acid with SiO_2_ surface (loading rate of 4.4 ± 0.8 nN s^−1^ for Asp and 4.6 ± 0.5 nN s^−1^ for Glu, in Tris buffer (50 mM, pH = 7.2)).

The adsorption of these two peptides to the silica surface could be explained by the strong binding interaction of the dicarboxylate moiety with the charged metal oxide surface, leading to the formation of a supramolecular functional coating by the self-assembly of these two low molecular weight peptides. From the contact angle measurement, it was observed that the hydrophobicity of the PA1 coated surface was moderately higher compared to that of the PA2 coated surface. This could be explained by the difference in the hydrophobicity scale between valine and leucine (*K*_d_ hydrophobicity for Val = 4.2 and Leu = 3.8) and the nature of the binding interaction or adhesive property of different anchoring amino acid residues in these peptides (Asp in PA1 and Glu in PA2) with the examined surface. In this context, we also quantitatively measured the interaction between the anchoring amino acids, Asp and Glu, with a well-defined silica (SiO_2_) surface using atomic force microscopy (AFM). For this purpose, the gold AFM tip was coupled with the examined amino acids through poly(ethylene glycol) 2-mercaptoethyl ether acetic acid (COOH-PEG-SH) (Fig. 3A). After the chemical modification of the AFM tip, the SiO_2_ surface was wetted with Tris buffer (50 mM, pH = 7.2) and the rupture forces from the surface were measured. We used the successful binding events (*n* ≈ 75–140) represented by force–distance (*F*–*D*) curves to construct histograms and applied a Gaussian fit to these histograms to calculate the average adhesion force. The results showed that the average adhesion force or most probable force (MPF) for aspartic acid is higher (142 ± 27 pN) (Fig. 3B) compared to glutamic acid (91 ± 18 pN) (Fig. 3C); this reflects the stronger adhesion of aspartic acid compared to glutamic acid with a silica (SiO_2_) surface. Based on the results obtained, we can assume that a peptide with aspartic acid as an anchoring moiety will exhibit better binding and adhere more strongly onto the silica surface compared to the peptide PA2 with glutamic acid as the anchoring unit.

In order to confirm the adsorption of peptides (PA1 and PA2) on the silica substrates, we performed attenuated total reflectance Fourier transform (ATR-FTIR) spectroscopy. A silica substrate coated with peptides PA1 and PA2 exhibits characteristic peaks in the region 1700–1800 cm^−1^. The substrate coated with PA1 exhibited two peaks at 1764 cm^−1^ and 1787 cm^−1^, and exhibited one broad peak at 1776 cm^−1^ while coated with PA2 (Fig. S11, ESI†). No such characteristic peak was found for the bare silica surface in this particular region. The peaks that appeared for the peptide-coated surface are very much similar to the characteristic peak value of a Si–acetoxy bond (Si–O–CO–).^36^ Therefore, the appearance of these peaks in the 1700–1800 cm^−1^ region suggests that the peptides are bound to the substrate through the carboxylate anchoring unit.

**Fig. 4:**
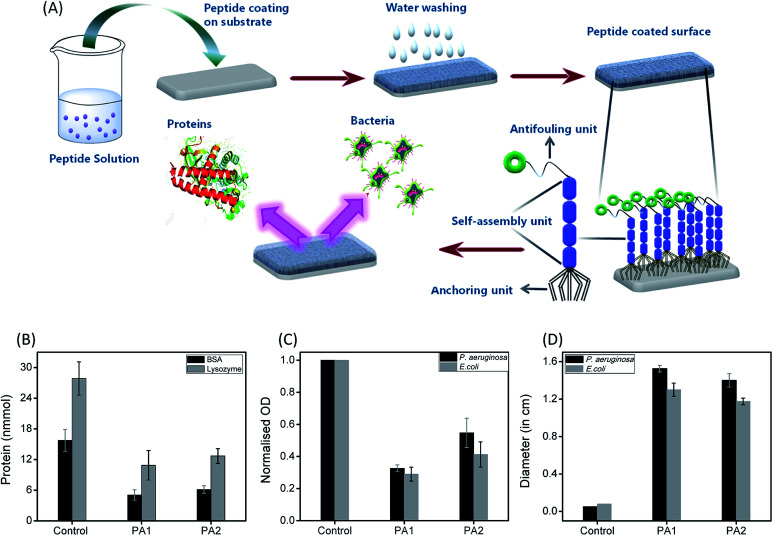
(A) Formation of antifouling coating based on the self-assembly of peptides: illustration represents the formation of a supramolecular coating on the desired substrate by the self-assembled tripeptides (PA1, PA2, having three different units; anchoring unit, self-assembly unit and antifouling moiety) exhibiting antifouling activity. Evaluation of the antifouling activity of the studied peptides (PA1, PA2); (B) adsorbed amounts of BSA (black) and lysozyme (grey) to bare (control) and coated silica surface with peptides PA1 and PA2. (c) Normalized optical density quantification of the accumulated *P. aeruginosa* (black) and *E. coli* (grey) on bare (control) and PA1 and PA2-coated silica surfaces. (D) Zone of inhibition of *P. aeruginosa* (black) and *E. coli* (grey) after 24 h incubation of surface coated with PA1 and PA2 as compared to the bare surface (control). The error bars represent the standard deviation (*n* = 5).

The formation of a bacterial biofilm onto any surface is usually preceded by the initial adsorption of bioorganic matter, which mediates the subsequent attachment of microorganisms.^1^ Therefore, we examined the resistant property of these peptide (PA1 and PA2) coated surfaces to protein adsorption. The uncoated (control) as well as peptide PA1 and PA2 coated silica substrate were incubated in a protein solution of both bovine serum albumin (BSA) and lysozyme for 3 hours at 37 °C at a concentration of 150 mM. To determine the adsorbed amounts of the proteins on the substrates with or without coating, we used the Non-interfering protein assay™ kit. The plot in Fig. 4B shows the adsorbed amounts of proteins onto bare and peptide-coated silica substrates. As seen in the results, both BSA and lysozyme were adsorbed onto the bare silica substrates (control). However, the substrate coated with either of the peptides showed that the amounts of proteins adsorbed were reduced considerably as compared to the amount of protein adsorbed onto the bare substrate. As shown in Fig. 4B, a greater reduction was observed in lysozyme adsorption with the coated substrate. In addition, comparison between the two peptides (PA1 and PA2) did not reveal any significant difference in their ability to resist protein adsorption. Overall, these results clearly demonstrate the ability of both the peptide-based coatings to resist protein adsorption and predictively exhibit antifouling activity.

To determine the effectiveness of the newly synthesized peptides to develop bacteriophobic implant coatings, we first investigated the potential ability of both the peptides (PA1 and PA2) to inhibit bacterial growth. The Kirby–Bauer disk diffusion assay was modified and performed to evaluate the ability of these peptides to inhibit the growth of two separate Gram-negative organisms *Pseudomonas aeruginosa* and *Escherichia coli*. As shown in Fig. 4D (ESI with a representative zone of inhibition image, Fig. S12, ESI†), incubation with both the peptides resulted in a distinct zone of inhibition averaging around 14 to 16 mm for *P. aeruginosa* and 12 to 13 mm for *E. coli*, indicating that both the peptides are bacteriostatic in nature. Interestingly, incubation (after 16 hours) with PA1 resulted in a greater zone of inhibition. In this context, we propose that the greater adhesion force of aspartic acid compared to glutamic acid make the binding affinity of PA1 higher compared to that of PA2, leading to a more hydrophobic coating as well as exhibiting a greater zone of inhibition.

The next aim was to assess the potential ability of these peptide-based functional coatings to prevent bacterial biofilm formation on silica surfaces. For this purpose, the uncoated substrate (control) and peptide (PA1 and PA2) coated surfaces were incubated in inoculums of *P. aeruginosa* and *E. coli* for 9 and 96 hours, respectively. These incubation times are permissible for the establishment of a biofilm by different bacterial strains. The incubated substrates were then stained with 2% (w/w) crystal violet (CV)^37^ and the amount of biofilm formed was determined by measuring the absorbance of the extracted CV dye from the bacteria.^38^ The absorbance of the CV dye is directly proportional to the number of bacteria adhered to the surface. As shown in Fig. 4C and S13, ESI,† both the peptides led to a decrease in the biofilm density as measured by the reduction of the amount of CV staining. In line with the results of the bacteriostatic effect, coating with PA1 seemed more effective in its antifouling activity compared to PA2. In fact, we observed a reduction of ∼70% (for PA2, ∼60%) in the amount of CV when the silica surface was coated with PA1 and incubated with *E. coli* in comparison to the bare surface (control). For the surface coated with peptide PA1 and incubated with *P. aeruginosa*, a decrement of 67% (for PA2 ∼45%) in the amount of CV was recorded (Fig. 4C). Therefore, the above results clearly indicate that a silica substrate coated with peptide PA1 with aspartic acid as the anchoring moiety exhibits a better antifouling property. This result agrees with our expectations based on the force measurements by AFM and contact angle measurements.

We have additionally synthesized a reference compound PA3 (PFB-VVE), which has a similar self-assembly and antifouling moiety as PA1, with the only difference being in the surface anchoring unit. PA3, unlike PA1, has glutamic acid as the surface binding unit. Further, we examined the resistance of a PA3-coated silica surface to protein adsorption (incubated in a protein solution of both bovine serum albumin (BSA) and lysozyme for 3 hours at 37 °C). The PA3-coated substrate exhibited a better response in resisting protein adsorption when compared to the bare surface (Fig. S18A, ESI†). More importantly, the antifouling assay demonstrated that the surface coated with PA1 is more bacteriophobic and seemed more effective in its antifouling activity compared to PA3 (Fig. S18B, ESI†). This result would be probably due to the better binding affinity of aspartic acid on the desired surface compared to glutamic acid, leading to better antifouling property due to the stronger binding of PA1 compared to PA3. Although this observation is pre-emptive in evaluating the exact effect of the antifouling and self-assembly unit in the peptide backbone in contributing to antifouling property. Our further studies with newly synthesized peptides with all the probable variations in the self-assembly and surface binding units along with theoretical calculations will try to provide detailed comparison and proper insights regarding the effect of both self-assembly and anchoring units on antifouling activity.

The eventual cytotoxicity of both the peptides was tested in human embryonic kidney cells (HEK 293 cells) by conventional MTT assays.^39^ Cell proliferation was estimated after 24 h of incubation following a standard protocol. As shown in Fig. S19A, ESI,† there were no substantial differences in the cell proliferation observed at concentrations ranging from 1 to 100 μM, indicating that compounds PA1 and PA2 are biocompatible. The estimated cellular viability was higher for PA1 (∼88–73%) as compared to PA2 (∼79–63%) at a concentration range of ≤10 to 100 μM (Fig. S19A, ESI†). The viability was reconfirmed using the trypan blue dye exclusion method in which the number of viable cells was counted by microscopic visualization^40^ (Fig. S19B, ESI†). The percentage viability calculated also corroborates the data obtained from the MTT assay.

## Conclusions

We have reported the synthesis and characterization of a novel class of two antifouling peptides (PA1 and PA2). The design of these peptides comprises three basic parts: Val–Val/Leu–Leu as the self-assembly unit, glutamic acid/aspartic acid with a dicarboxylate group as the surface affixing unit and a penta-fluorinated aromatic moiety as the antifouling unit. Both the peptides form functional supramolecular coatings on the silica surface that are able to resist biofilm formation. The results obtained clearly demonstrate that peptide PA1 with aspartic acid as the anchoring moiety demonstrated a better antifouling property along with the formation of a more hydrophobic coating on the examined surface. This result is supported by the greater adhesion force value of aspartic acid compared to glutamic acid with the silica surface as evident by the force measurement analysis using AFM. These newly designed peptides can find several applications in aspects of biomedical research, water treatment and marine fouling. The methodology described, including a simple drop-coating method, can avoid the synthetic complications as well as multi-step nature of the process involved with surface modification, thus making this procedure cost-effective with a promising outlook for commercialization. In addition, this opens the possibility of using medical equipment coated with these antifouling peptides to prevent infections. Most importantly, this result can serve the basis for providing more detailed explorations of design and chemical composition that involve the dicarboxylate group as the substrate binding unit, which may provide more efficient and smart antifouling materials and further insights into the mechanism of antifouling activity.

## Experimental section

### Materials and methods

All chemicals and solvents are commercially available and were used as received without further purification. Amino acids such as valine, leucine, glutamic acid, aspartic acid and *N*,*N*′-dicyclohexyl-carbodiimide, di-*tert*-butyl dicarbonate, 1-hydroxybenzotriazole, trimethylchlorosilane, *o*-(benzotriazole-1-yl)-*N*,*N*,N′,*N*′-tetramethyluronium hexafluorophosphate and triethylamine were purchased from Sisco Research Laboratories (SRL) Pvt. Ltd. Potassium hydrogen sulfate was purchased from Loba Chemie Pvt. Ltd. Trifluoroacetic acid was purchased from Finar Ltd. 2,3,4,5,6-Pentafluorobenzaldehyde was purchased from Sigma Aldrich. 1,1,1,3,3,3-Hexafluoro-2-propanol (HFP) was purchased from Tokyo Chemical Industry Co. Ltd. Luria-Bertani Agar and Broth (LB-Agar and Broth) were purchased from Himedia Laboratories. Poly(ethylene glycol) 2-mercaptoethyl ether acetic acid, crystal violet solution, acetic acid and bovine serum albumin were purchased from Sigma-Aldrich (Merck). Lysozyme was purchased from Thermo Fischer Scientific. A stock of the HEK 293 cell line was purchased from the National Cell Line Repository, National Centre for Cell Science Complex, Pune – 411007, India and maintained in culture.

### Self-assembly of PA1 and PA2

A fresh stock solution of peptides was prepared by dissolving the lyophilized forms of the PA1 and PA2 in HFP to a concentration of 100 mg mL^−1^. Then, we blended these peptide solutions in several different proportions and diluted them with aqueous ethanol to get the desired concentrations of these peptides for self-assembly. The polarized solvent allowed the molecules to self-assemble.

### High-resolution scanning electron microscopy (HR-SEM)

A 10 μL drop of a self-assembled solution of PA1 and PA2 was placed on a glass cover slip and allowed to dry at RT. The substrates were then coated with gold using a Leica EM ACE200 2–3 nm gold coater. SEM analysis was performed using a high-resolution scanning electron microscope (FE-SEM, JEOL JSM-7100F) operating at 18 kV.

### Microanalysis

(C, H, and N) analysis was performed using a Vario Micro Cube (Elementar) instrument.

### Atomic force microscopy analysis

Topography images of the self-assembled structures (formed by PA1 and PA2) on the glass cover slips were taken using a NT-MDT MOSCOW instrument (model Ntegra Aura) working in AC mode. Si_3_N_4_ cantilever probes with a spring constant of 3 Nm^−1^ and a resonance frequency of 75 kHz were used.

### Fourier transform infrared spectroscopy (FT-IR)

Fourier transform infrared spectra were recorded using an IR Tracer-100 FT-IR spectrometer (Shimadzu) with a Deuterated Lanthanum α Alanine doped TriGlycine Sulphate (DLaTGS) detector. The peptide self-assembled solutions were deposited on a CaF_2_ window and dried under vacuum. The peptide deposits were resuspended with D_2_O and subsequently dried to form thin films. This suspension procedure was repeated twice to ensure maximal hydrogen-to-deuterium exchange. The measurements were taken using 4 cm^−1^ resolution and an average of 2000 scans. The transmittance minimal values were determined using the Lab solutions IR analysis program (IR Tracer).

### Tip functionalization

The chemical modification of the AFM tip was done following the process described previously.^22*a*,41^ The AFM cantilevers were cleaned by dipping them in ethanol for 20 minutes. After drying at room temperature, the tips were immersed in a solution of poly(ethylene glycol) 2-mercaptoethyl ether acetic acid (COOH-PEG-SH; MW 3500) at a concentration of 5 mM in chloroform for 1 hour at room temperature. The tips were then extensively washed with chloroform and DMF. The carboxylate groups of the attached PEG molecule were then coupled with the amine group of the desired C-termini-protected amino acid. Coupling was done using C-termini-protected amino acid/diisopropylethylamine/HBTU with a molar ratio of 1 : 1 : 1 and at a total concentration of 30 mM in 5 mL NMP for 2 hours. Then, the tips were dissolved in a methanolic solution of 5 mM NaOH (Fig. S10, ESI†). Finally, the amino acid functionalized tips were repeatedly washed with NMP, DMF, chloroform, ethanol and water, and then dried in air.

### Force spectroscopy measurements

Force spectroscopy measurements were carried out using PBS buffer (50 mM, pH = 7.4) at 298 K, using a Molecular Force Probe 3D Origin AFM system (MFP 3D Origin Asylum research). The AFM cantilevers, with a spring constant ranging from 0.01–0.06 Nm^−1^, were calibrated using the thermal fluctuation method (included in the AFM software) with an absolute uncertainty of ∼10%. Measurements were obtained by bringing the amino acid-functionalized tip to the surface until it was brought into contact with the surface, with a compression force of ∼200 pN and then immediately retracting the tip at a speed of 0.4 μm s^−1^, for a distance of ∼200 nm. During the retraction, constant force plateaus were observed in the force–distance (*F*–*D*) curves.

### Data analysis

Prior to analysis, the deflection values (V) were converted to force by multiplying the photodiode sensitivity (V m^−1^) using the experimentally determined spring constant.^42^ To calculate the apparent loading rate, we fitted at least 30 force *vs.* distance curves with the worm-like chain (WLC) model just prior to ruptures, which were then used for preparing histograms of the apparent loading rates. The unbinding forces between the amino acids and silica surface were derived from the jump in force following the separation of the cantilever from the substrate. This was done using the data processing software.

### Contact angle measurement

Contact angle measurements were carried out using a substrate based analyzer at the solid/water interface (Model No: HO-IAD-CAM-01, Holmarc, Opto-Mechatronics Pvt. Ltd). Each experimental measurement consisted of three repeats, and the reported angles were averaged.

### Preparation of substrates

Silica substrates of 1 cm^2^ were first washed thoroughly for 2 minutes each with acetic acid, acetone, 100% ethanol, followed by 70% ethanol before autoclaving them. Peptide solutions (1–5 mg mL^−1^) were dropped on to the substrate, dried properly, washed with deionised water, and kept in a clean Petri dish and incubated for 4 hours at RT. The slides were rinsed and used.

### Biofilm formation

*Pseudomonas aeruginosa* and *Escherichia coli* were grown in LB medium respectively overnight at 37 °C in loosely capped tubes with agitation (120 rpm) to the stationary phase. Then, cultures were diluted to 10^8^ CFU mL^−1^ with LB and grown until the mid-log phase. 2 mL of the mid-log phase culture was transferred to a well of a 6-well plate in which the substrates were placed horizontally. This was incubated at 37 °C for 9 hours for the formation of a biofilm by *P. aeruginosa* and 96 hours for the formation of a biofilm by *E. coli*. Every 5 hours the medium was replaced with a fresh one to ensure a sufficient supply of nutrients.

### Crystal violet assay

The biofilm assay was adapted from G. A. Toole *et al.* in 2011.^43^ Briefly, after incubation with the bacteria as mentioned above, the peptide-coated substrates were gently rinsed 3 times and stained with 0.1% crystal violet at room temperature for 20 minutes. The stained samples were rinsed 3–4 times with water and left to dry in air. Eventually, the bound dye was eluted with 30% acetic acid. Absorbance values were recorded at 550 nm in a microplate reader (Biorad) using 30% acetic acid in water as the blank. All measurements were performed five times and averaged.

### Protein adsorption assay

50 μL of a single protein solution of BSA and lysozyme (150 μM in PBS) was applied onto the peptide coated substrates in a Petri dish. The plate was placed in a humidified incubator at 37 °C for 3 hours. The substrates were then rinsed 3 times with PBS (pH = 7.43, 10 mM, 150 mM NaCl) and transferred into test tubes with 1 mL of 1.0% (w/w) SDS. The samples were shaken for 60 minutes and sonicated for 20 minutes at room temperature to detach the adsorbed proteins. Protein concentrations in the SDS solution were determined using the Non-interfering protein assay (Merck, Millipore) according to the instructions of the manufacturer using a microplate reader at 480 nm (Biorad). All measurements were performed five times and averaged.

### Agar well diffusion method

The agar well diffusion assay used was a modification of Perez *et al.* (1990).^44^ Briefly, 0.2 mL of diluted inoculums (2 × 10^8^ CFU mL^−1^) of *E. coli* and 0.25 mL (2 × 10^8^ CFU mL^−1^) *P. aeruginosa* were spread on LB agar plates. Wells with a 6 mm diameter were punched into the agar and filled with 10 μL of the peptide solutions at a concentration of 0.5 mg mL^−1^ and only the solvent blank (ethanol) separately. The plates were incubated at 37 °C overnight. The zone of inhibition of test organism growth around each well was measured in mm. Each test was carried out five times and the results were averaged.

### MTT assay

HEK 293 cells (human embryonic kidney 293 cells are a specific cell line originally derived from human embryonic kidney cells) were cultured in a 25 cm^2^ flask at 37 °C and 5% CO_2_ in a humidified atmosphere in complete MEM supplemented with 10% FBS and 4 mm l-glutamine. For the experiment, cells were seeded in a 96-well plate at a density of 50 000 cells, grown to 70–80% confluency and incubated with peptide in an appropriate serum-free medium for 24 h under standard growth conditions. Then, 10 μL of MTT solution (5 mg mL^−1^, Sigma) was added to each well and incubated at 37 °C in 5% CO_2_ for 4 hours. After centrifugation at 3000*g* for 15 minutes, the supernatant was removed and 150 μL of DMSO (dimethylsulfoxide, Sigma) was added to dissolve the formazan crystals. The absorbance was measured at 570 nm using a microplate reader. Ethanol was used as a solvent control and cisplatin (100 μg mL^−1^) was used as a positive control.

### Trypan blue dye exclusion assay

The cell membrane integrity of HEK 293 cells treated with the peptides obtained from the 24 hours assay (as above) was analysed using trypan blue. Pelleted cells were suspended in 100 μL of RMPI medium and 20 μL of cell suspension was mixed with equal volume of the trypan blue solution (0.4% in PBS; Sigma). After 5 minutes of incubation at room temperature, the cells were counted in a Neubauer Improved hemocytometer. The percentage of cells not stained blue, that is with an intact cell membrane, was calculated. Ethanol was used as a solvent control and cisplatin (100 μg mL^−1^) was used as a positive control. The percentage viability was calculated by dividing the number of viable cells by the number of total cells and multiplying by 100.

## Conflicts of interest

There are no conflicts of interest to declare.

## Supplementary Material

RA-010-C9RA10018K-s001
