# Supplementary material for: The design and development of short peptide-based novel smart materials to prevent fouling by the formation of non-toxic and biocompatible coatings
Source: RSC Adv. 2020 Apr 1;10(23):13420–9. doi: 10.1039/c9ra10018k (PMC9051384; doi:10.1039/c9ra10018k)
Supplement: RA-010-C9RA10018K-s001 [file RA-010-C9RA10018K-s001.pdf]

Electronic Supplementary Material (ESI) for RSC Advances.
This journal is © The Royal Society of Chemistry 2020

Supplementary Information

Design and Development of Short Peptide Based Novel Smart Material to Prevent Fouling by the Formation of Nontoxic and Biocompatible Coating

Amutha Arul,[a] Subramaniyam Sivagnanam,[a] Ananta Dey,[b][c] Oindrilla Mukherjee, [d] Soumyajit Ghosh,* [a] and Priyadip Das*[a]*

(a) *Department of Chemistry, SRMIST, SRM Nagar, Potheri, Kattankulathur, Kancheepuram District, Chennai, Tamil Nadu, 603203, India.*

(b) *Academy of Scientific and Innovative Research (AcSIR), Ghaziabad – 201002, India*

(c) *CSIR-Central Salt & Marine Chemicals Research Institute, Bhavnagar 364002, India*

(d) *Department of biotechnology, National Institute of Technology, Durgapur, West Bengal-713209, India*

* *Email: priyadipcsmcri@gmail.com, soumyajitghosh89@gmail.com*

Experimental section

Peptide synthesis: Peptides were synthesized by conventional solution-phase methods. Peptide coupling was mediated by coupling reagents dicyclohexylcarbodiimide and 1-hydroxybenzotriazole (DCC/HOBt). The products were purified by column chromatography using silica gel (100–200 mesh) as the stationary phase and an n-hexane–ethyl acetate mixture as an eluent. The final compounds were fully characterized by Bruker 500 MHz ^{1}H-NMR spectroscopy and Mass spectroscopy (Shimadzu, Japan, LCMS-2020 Spectrometer).

A. Synthesis of Peptide PA1

Synthesis of BOC-NH-Val-OH (A1): A solution of L-valine (3 g, 25.63 mmol) in a mixture of dioxane (40 mL), water (20 mL), and 1 M NaOH (22 mL) was stirred and cooled in an ice-water bath. Di-tert-butyl dicarbonate (6.98g, 31.98 mmol) was added and stirred continuously at room temperature (RT) for 6h. Then, the solution was concentrated under vacuum using rotary evaporator to about 10–15 mL, cooled in an ice-water bath, covered with a layer of ethyl acetate (about 50 mL), and acidified with a dilute solution of $KHSO_4$ to pH 2–3 (determined by congo

red). The aqueous phase was extracted with ethyl acetate and this operation was performed repeatedly. The ethyl acetate extracts were pooled, washed with water, dried over anhydrous Na_2SO_4 and evaporated under vacuum. The pure material was obtained as a waxy solid. Yield: 5.41 g (24.87 mmol, 97.18%) (Scheme S1).

Synthesis of NH_2-Val-OMe Hydrochloride (A2): L-valine 4g (34.17 mmol) was taken in a round bottom flask and dissolved in 80 mL MeOH. Then, 8.6mL (67.76 mmol) of Trimethyl chlorosilane (TMSCl) was added to the resulting solution slowly in a drop wise manner and stirred for 8h at room temperature. After the completion of reaction (as monitored by TLC), the excess solvent was evaporated on a rotary evaporator to get the solid desired product L-Valine methyl ester hydrochloride. Yield: 5.52g (32.93 mmol, 96.38%) (Scheme S1).

Synthesis of BOC-NH-Val-Val-OMe (A3): 5.41 g (24.87 mmol) of Boc-NH-Val-OH was dissolved in 40mL dry DCM in an ice-water bath. NH_2-Val-OMe.HCl 5.40 g (32.23 mmol) and Et_3N 5ml (35.87mmol) were then added to the reaction mixture, followed immediately by the addition of 6.14g (29.75mmol) dicyclohexylcarbodiimide (DCC) and 4.55g (29.75 mmol) of HOBt. The reaction mixture was allowed to warm-up to RT and was stirred for 48 h. DCM was evaporated and the residue was dissolved in ethyl acetate (50 mL). The dicyclohexylurea (DCU) was filtered off. The organic layer was washed with 2M HCl (3 X 50 mL), brine (2 X50 mL), 1M sodium carbonate (3 X 50 mL), and brine (2 X 50 mL), and finally dried over anhydrous sodium sulfate. It was then evaporated using vacuum to yield Boc-NH-Val-Val-OMe as a white solid. The product was purified by silica gel (100–200 mesh) using n-hexane–ethyl acetate (3:1) as eluent. Yield: 5.69 g (17.22 mmol, 67.25%). ^{1}H NMR ($CDCl_3$, 500 MHz, δ ppm): 6.29 (b, 1H, NH), 4.98(b, 1H, NH), 4.49-4.47 (dd, 1H, C_αH, Val), 3.85-3.82 (m, 1H, C_αH, Val) 3.67 (s, 3H, OMe), 2.13-2.09 (dd, 2H, C_βH, Val), 1.38 (s, 9H, Boc), 0.91-0.84 (m, 12H, CH_3, Val). ESI-MS (m/z): $[M+Na]^+$ = 353.42 (calculated); 353.48 (observed) (SchemeS1).

Synthesis of BOC-NH-Val-Val-OH (A4): To 5.69 g (17.22 mmol) of Boc-NH-Val-Val-OMe, 40 mL MeOH and 2M 20 mL NaOH were added and the progress of saponification was monitored by thin layer chromatography (TLC). The reaction mixture was stirred and after 10h, the methanol was removed under vacuum; the residue was dissolved in 50 mL of water, and

washed with diethyl ether (2 X 50 mL). Then, the pH of the aqueous layer was adjusted to 2 using 1M HCl and extracted with ethyl acetate (3 X 50 mL). The combined organic layers were pooled, dried over anhydrous sodium sulfate, and evaporated under vacuum to obtain the desired compound as a waxy solid. Yield: 5.32 g (16.81 mmol, 97.65%). ^{1}H NMR ($CDCl_3$, 500 MHz, δppm): 6.89-6.87(d, 1H, NH), 5.32-5.30(b, 1H, NH), 4.53-4.51 (dd, 1H, $C_{\alpha}H$, Val), 4.06-3.91 (m, 1H, $C_{\alpha}H$, Val) , 2.20-1.98 (m, 2H, $C_{\beta}H$-Val), 1.36 (s, 9H, Boc),0.90-0.85 (m, 12H, CH_3, Val). ESI-MS (m/z): $[M-H]^+$ = 315.39 (calculated); 315.15 (observed) (SchemeS1).

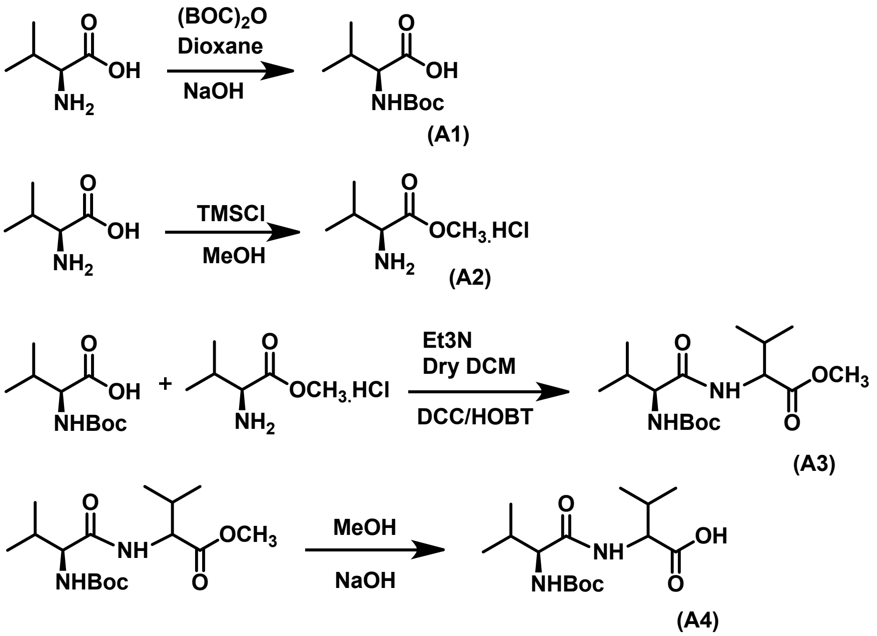

Scheme S1: Synthetic methodologies adopted for the synthesis of **A1, A2, A3** and **A4**.

Synthesis of NH_2-Asp-$(OMe)_2$ Hydrochloride (A5): L-aspartic acid 4g (30.05 mmol) was taken in a round bottom flask and dissolved in 80 mL MeOH. Then, 8.6mL (67.76 mmol) of Trimethyl chlorosilane (TMSCl) was added to the resulting solution slowly in a drop wise manner and stirred for 8h at room temperature. After the completion of reaction (as monitored by TLC), the excess solvent was evaporated on a rotary evaporator to get the solid desired product L-Aspartic acid methyl ester hydrochloride. Yield: 5.56g (28.13 mmol, 93.79%) (Scheme S2).

Synthesis of BOC-NH-Val-Val-Asp-$(OMe)_2$ (A6) : 5.32g (16.81 mmol) of BOC-NH-Val-Val-OH was dissolved in 50mL dry DCM in an ice-water bath. NH_2-Asp-$(OMe)_2$.HCl 4.483 g

(22.68 mmol) and Et_3N (5 ml,35.87 mmol) were then added to the reaction mixture, followed immediately by the addition of 4.16 g (20.17mmol) dicyclohexylcarbodiimide (DCC) and 3.08 g (20.17 mmol) of HOBt. The reaction mixture was allowed to warm-up to RT and was stirred for 48 h. DCM was evaporated and the residue was dissolved in ethyl acetate (45 mL). The dicyclohexylurea (DCU) was filtered off. The organic layer was washed with 2M HCl (3 X 50 mL), brine (2 X50 mL), 1 M sodium carbonate (3 X 50 mL), and brine (2 X 50 mL), and finally dried over anhydrous sodium sulfate. It was then evaporated using vacuum to yield Boc-NH-Val-Val-Asp-$(OMe)_2$ as a white solid. The product was purified by silica gel (100–200 mesh) using n-hexane–ethyl acetate (3:1) as eluent. Yield: 4.84 g (10.53 mmol, 62.68%). ^{1}H NMR ($CDCl_3$, 500 MHz, δ ppm): 6.90-6.88(d, 1H, NH), 6.56-6.54 (d, 1H, NH), 5.03-5.02 (d, 1H, NH), 4.81-4.79 (d, 1H, , $C_\alpha H$, asp),4.28-4.25(m, 1H, , $C_\alpha H$, val) 3.87-3.85 (t, 1H, $C_\alpha H$, val) 3.68 (s, 3H, -OMe), 3.63 (s, 3H, OMe) ,2.99-2.94 (dd, 1H, CβH, val) 2.77-2.72 (dd, 1H, $C_\beta H$, val) 2.11-2.06 (dd, 2H, $C_\beta H$, asp) 1.37 (s, 9H, Boc) 0.91-0.85(m,12H, CH_3). ESI-MS (m/z): $[M+Na]^+$= 482.53 (calculated); 482.87 (observed) (SchemeS2).

Synthesis of NH_2-Val-Val-Asp-$(OMe)_2$ (A7): 4.84 g (10.53 mmol) of Boc-NH-Val-Val-Asp-$(OMe)_2$ was dissolved in 40 mL of DCM in an ice bath. Then, 8 mL of TFA was added and stirred for 2h. The progress of the reaction was monitored by TLC. After the reaction was completed, all solvents were evaporated in a rotary evaporator. The product was then dissolved in water, neutralized with $NaHCO_3$ solution, extracted with ethyl acetate, dried over anhydrous sodium sulfate and evaporated by rotary evaporator to obtain an oily product, which was immediately used for the next reaction. Yield: 3.53 g (9.82 mmol, 93.38%).^{1}H NMR (DMSO-d_6, 500 MHz, δ ppm): 4.59 (s, 1H, $C_\alpha H$, asp),4.15-4.09(dd, 1H, $C_\alpha H$, val) 3.57-3.49 (d, 6H, OMe) 3.31-3.21 (d, 1H, $C_\alpha H$, val) ,2.82-2.79 (d, 1H, CβH, val) 2.71-2.64 (m, 1H, $C_\beta H$, val) 1.95(S, 2H, $C_\beta H$, asp) 0.87-0.80(m,12H, CH_3). ESI-MS (m/z): $[M+H]^+$= 360.42 (calculated); 360.10 (observed).

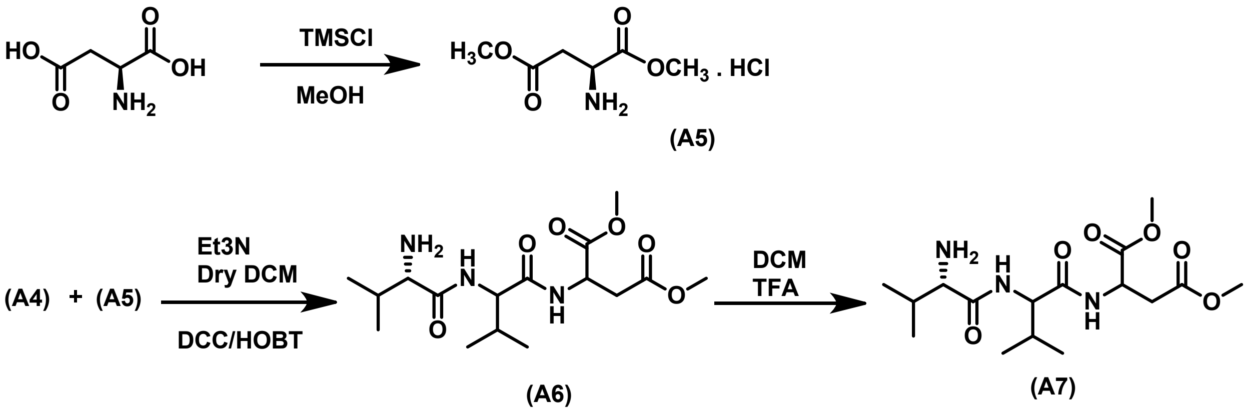

Scheme S2: Synthetic methodologies adopted for the synthesis of **A5, A6** and **A7.**

Synthesis of PFB-NH-Val-Val-Asp-$(OMe)_2$ (A8): 3.0 gm (8.34 mmol) Of NH_2-Val-Val-Asp-$(OMe)_2$ was dissolved in 40 ml of methanol. Then 2,3,4,5,6-Pentafluoro benzaldehyde (1.79g,9.12mmol) added to the methanolic solution of A7. The resulting mixture was stirred for 8h. After the reaction was completed (confirmed by TLC) white solid precipitate appeared. This precipitate was filtered off and washed with n-hexane and cold methanol and dried in desiccator to afford PFB-NH-Val-Val-Asp-$(OMe)_2$ (A8) as a pure product. Yield: 2.71g (5.04 mmol, 60.4%). ^{1}H NMR ($CDCl_3$, 500MHz, δppm): 8.23 (s,1H, PFB) 7.16-7.14 (d, 1H, NH), 6.75-6.74 (d, 1H, NH), 4.74-4.71 (dd, 1H, C_αH, asp),4.30-4.28(d, 1H, C_αH, val) 3.65 (s, 3H, OMe), 3.58 (s, 3H, OMe)),2.95-2.91 (dd, 1H, C_βH, val) 2.74-2.68 (dd, 1H, C_βH, val), 2.33-2.17 (d, 2H, C_βH, asp) 0.98-0.87(m,12H, CH_3). ESI-MS (m/z): $[M+H]^+$ =538.48 (calculated); 538.05 (observed) ; $[M+Na]^+$=560.48 (calculated); 560.15 (observed); $[M+2Na]^+$ = 583.48 (calculated); 583.05 (observed) (SchemeS3).

Synthesis of PFB-NH-Val-Val-Asp-$(OH)_2$ (PA1): 2g (3.72 mmol) of PFB-NH-Val-Val-Asp-$(OMe)_2$ (A8) was dissolved in 30 mL MeOH. Then 2M 15 mL NaOH was added and the progress of saponification was monitored by thin layer chromatography (TLC). The reaction mixture was stirred for 10h, the methanol was removed under vacuum; the residue was dissolved in 50 mL of water, and washed with diethyl ether (2 X 50 mL). Then, the pH of the aqueous layer was adjusted to 2 using 1M HCl and extracted with ethyl acetate (3 X 50 mL). The collected organic layer pooled and dried over anhydrous sodium sulfate, and evaporated under vacuum to obtain the desired di-carboxy compound as a semi solid. Yield: 1.35 g (2.65 mmol, 71.42%). ^{1}H NMR ($CDCl_3$, 500MHz, δppm): 10.21 (s,2H, Asp-COOH) , 8.24 (s,1H, PFB,

CH=N) 7.16 (d, *J*=9 Hz, 1H, NH), 6.75 (d, *J*=8 Hz, 1H, NH), 4.75-4.72 (m, 1H, $C_{\alpha}H$, Asp),4.31-4.29 (dd, 1H, $C_{\alpha}H$, val) 3.64 (d, 1H, $C_{\alpha}H$, Val), 2.96-2.92 (m, 1H, $C_{\beta}H$, CH_2-Val)),2.75-2.69 (m, 1H, $C_{\beta}H$, CH_2-Asp) 2.36-2.29 (m, 1H, $C_{\beta}H$, CH-Val), 2.23-2.16 (m, 1H, $C_{\beta}H$,CH_2-Val) 0.99-0.88 (m,12H, CH_3), ESI-MS (m/z): $[M-H]^+$ = 508.42 (calculated); 508.21 (observed) (SchemeS3). Elemental analysis calcd (%): ($C_{21}H_{24}F_5N_3O_6$): C 49.51, H 4.75, N 8.25, O 18.84; found: C 48.89, H 5.05, N 8.03.

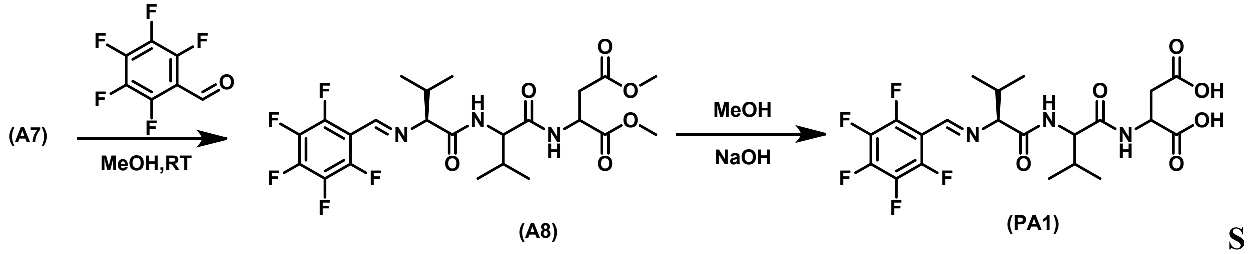

S

cheme S3: Synthetic methodologies adopted for the synthesis of **A8** and **PA1.**

B. Synthesis of Peptide PA2

Synthesis of BOC-NH-Leu-OH (B1): A solution of L-leucine (2g, 15.24 mmol) in a mixture of dioxane (30mL), water (15 mL), and 1 M NaOH (16 mL) was stirred and cooled in an ice-water bath. Di-tert-butyl dicarbonate (3.98 g, 18.23 mmol) was added and stirred continuously at room temperature (RT) for 6h. Then, the solution was concentrated using vacuum to about 10–15 mL, cooled in an ice-water bath, covered with a layer of ethyl acetate (about 50 mL), and acidified with a dilute solution of $KHSO_4$ to pH 2–3 (determined by congo red). The aqueous phase was extracted with ethyl acetate and this operation was performed repeatedly. The ethyl acetate extracts were pooled, washed with water, dried over anhydrous Na_2SO_4 and evaporated under vacuum. The pure material was obtained as a waxy solid. Yield 3.42 g (14.78 mmol, 97.03%) (Scheme S4).

Synthesis of NH_2-Leu-OMe hydrochloride (B2): L-leucine 3g (22.87 mmol) was taken in a round bottom flask and dissolved in 60 mL MeOH. Then, 6mL (47.27 mmol) of Trimethyl chlorosilane (TMSCl) was added to the resulting solution slowly in a drop wise manner and stirred for 8h at room temperature. After the completion of reaction (as monitored by TLC), the excess solvent was evaporated on a rotary evaporator to get the solid desired product L-Leucine methyl ester hydrochloride. Yield 3.97g (14.61 mmol, 95.78%) (Scheme S4).

Synthesis of BOC-NH-Leu-Leu-OMe (B3): 3.42 g (14.78 mmol) of Boc-NH-Leu-OH was dissolved in 40mL dry DCM in an ice-water bath. NH_2-Leu-OMe.HCl 3.83 g (21.08 mmol) and Et_3N 5ml (35.87 mmol) were then added to the reaction mixture, followed immediately by the addition of 3.86 g (18.73mmol) dicyclohexylcarbodiimide (DCC) and 2.86 g (18.73 mmol) of HOBt. The reaction mixture was allowed to warm-up to RT and was stirred for 48 h. DCM was evaporated and the residue was dissolved in ethyl acetate (45 mL). The dicyclohexylurea (DCU) was filtered off. The organic layer was washed with 2M HCl (3 X 50 mL), brine (2 X50 mL), 1 M sodium carbonate (3 X 50 mL), and brine (2 X 50 mL), and finally dried over anhydrous sodium sulfate. It was then evaporated using vacuum to yield Boc-NH-Leu-Leu-OMe as a white solid. The product was purified by silica gel (100–200 mesh) using n-hexane–ethyl acetate (3:1) as eluent. Yield: 3.26 g (9.09 mmol, 61.6%). ^{1}H NMR ($CDCl_3$, 500 MHz, δppm): 6.37-6.36 (d, 1H, NH),4.79-4.80 (d, 1H, C_αH, Leu),4.57-4.52 (td, 1H, C_αH, Leu), 4.03(b, 1H, NH), 3.66 (s, 3H, OMe), 1.61 (b, 4H, -CH2-Leu), 1.52-1.41(m, 2H, CH, Leu) 1.37 (s, 9H, Boc), 0.89-0.85 (dd, 12H, CH_3, Leu). ESI-MS (m/z): $[M+Na]^+$ = 381.47 (calculated); 381.28 (observed) (SchemeS4).

Synthesis of BOC-NH-Leu-Leu-OH (B4): To 3.26g (9.09 mmol) of Boc-NH-Val-Val-OMe, 40 mL MeOH and 2M 15 mL NaOH were added and the progress of saponification was monitored by thin layer chromatography (TLC). The reaction mixture was stirred for 10h and then the methanol was removed under vacuum; the residue was dissolved in 50 mL of water, and washed with diethyl ether (2 X 50 mL). Then, the pH of the aqueous layer was adjusted to 2 using 1M HCl and extracted with ethyl acetate (3 X 50 mL). The organic extracts were pooled, dried over anhydrous sodium sulfate, and evaporated under vacuum to obtain the desired compound as a waxy solid. Yield: 2.86 g (8.30 mmol, 91.34%). ^{1}H NMR ($CDCl_3$,500 MHz, δppm): 6.39-6.37(d, 1H, NH), 4.84-4.83 (d, 1H, NH), 4.54-4.50 (m, 1H, C_αH, leu), 4.05-4.03 (m, 1H, C_αH, leu), 1.61-1.55 (m, 4H, C_βH, leu) , 1.50-1.40 (m, 2H, CH, leu), 1.37 (s, 9H, Boc),0.88-0.85 (m, 12H, CH_3, leu). ESI-MS (m/z): $[M-H]^+$ = 343.45 (calculated); 343.40 (observed) $[2M-H]^+$ = 687.90 (calculated); 687.20 (observed) (SchemeS4).

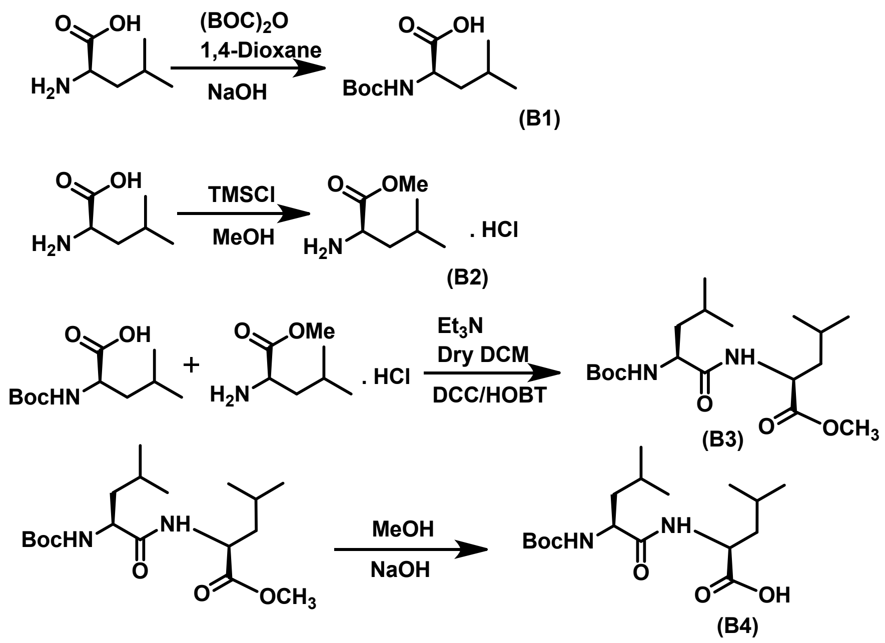

Scheme S4: Synthetic methodologies adopted for the synthesis of **B1, B2, B3** and **B4**.

Synthesis of NH_2-Glu-$(OMe)_2$ Hydrochloride (B5): L-glutamic acid 3g (20.40 mmol) was taken in a round bottom flask and dissolved in 50 mL MeOH. Then, 5mL (39.39 mmol) of Trimethyl chlorosilane (TMSCl) was added to the resulting solution slowly in a drop wise manner and stirred for 8h at room temperature. After the completion of reaction (as monitored by TLC), the excess solvent was evaporated on a rotary evaporator to get the solid desired product L-Glutamic methyl ester hydrochloride. Yield 3.78g (17.86 mmol, 87.70%) (SchemeS5).

Synthesis of BOC-NH-Leu-Leu-Glu-$(OMe)_2$ (B6) : 2.86 g (8.30 mmol) of BOC-NH-Leu-Leu-OH was dissolved in 40mL dry DCM in an ice-water bath. NH_2-Glu-$(OMe)_2$.HCl 2.37 g (11.21mmol) and Et_3N (4ml, 26.69 mmol) were then added to the reaction mixture, followed immediately by the addition of 2.05 g (9.96 mmol) dicyclohexylcarbodiimide (DCC) and 1.53g (9.96 mmol) of HOBt. The reaction mixture was allowed to warm-up to RT and was stirred for 48 h. DCM was evaporated and the residue was dissolved in ethyl acetate (45 mL). The dicyclohexylurea (DCU) was filtered off. The organic layer was washed with 2M HCl (3 X 50 mL), brine (2 X50 mL), 1 M sodium carbonate (3 X 50 mL), and brine (2 X 50 mL), and finally dried over anhydrous sodium sulfate. It was then evaporated using vacuum to yield Boc-NH-Leu-Leu-Glu-(OMe) $_2$ as a white solid. The product was purified by silica gel (100–200 mesh) using n-hexane–ethyl acetate (3:1) as eluent. Yield: 2.53 g (5.04 mmol, 60.81%).1H NMR

($CDCl_3$, 500 MHz, δ ppm): 6.76-6.74(d, 1H, NH), 6.44-6.43 (d, 1H, NH), 4.79-4.78 (d, 1H, NH), 4.52-4.48 (m, 1H, $C_\alpha H$, leu), 4.38-4.34 (m, 1H, $C_\alpha H$, leu),4.02(s, 1H, $C_\alpha H$, glu) 3.68 (s, 3H, -OMe), 3.60 (s, 3H, OMe) 2.36-2.11(d, 2H, $C_\beta H$, glu), 2.66-1.88(dd,2H, $CH_{2,}$glu) 1.65-1.58 (m, 4H, $C_\beta H$, leu) , 1.50-1.40 (m, 2H, CH, leu), 1.37 (s, 9H, Boc), 0.88-0.84(m, 12H, CH_3, leu) ESI-MS (m/z): $[M+H]^+$= 502.61(calculated); 502.35 (observed); $[M+Na]^+$ = 524.61(calculated); 524.15 (observed) (SchemeS5).

Synthesis of NH_2-Leu-Leu-Glu-$(OMe)_2$ (B7) : 2 g (3.98 mmol) of Boc-NH-Leu-Leu-Glu-$(OMe)_2$ was dissolved in 25 mL of DCM in an ice bath. Then, 4 mL of TFA was added and stirred for 2h. The progress of the reaction was monitored by TLC. After the completion of the reaction all solvents were evaporated in a rotary evaporator. The product was then dissolved in water, neutralized with $NaHCO_3$ solution, extracted with ethyl acetate, dried over anhydrous sodium sulfate and evaporated by rotary evaporator to obtain an oily product, which was immediately used for the next reaction Yield: 1.45 g (3.61 mmol, 91.19%).1H NMR ($CDCl_3$, 500 MHz, δ ppm): 8.24-8.23(d, 1H, NH), 7.55-7.53 (d, 1H, NH), 4.47-4.39 (q, 1H, $C_\alpha H$,leu), 4.35-4.31 (q, 1H, $C_\alpha H$,leu), 3.94-3.92 (t, 1H, $C_\alpha H$, glu) 3.65 (s, 3H, -OMe), 3.59 (s, 3H, OMe) 3.09-3.05(q, 2H, $C_\beta H$, glu), 2.33-2.30 (dd,2H, $CH_{2,}$glu) , 2.16-1.90 (m, 2H, $C_\beta H$, leu) , 1.67-1.61 (m, 2H, $C_\beta H$, leu), 1.56-1.55 (m, 2H, CH, leu), 0.86-0.79 (m, 12H, CH_3, leu). ESI-MS (m/z): $[M+H]^+$= 402.50 (calculated); 402.20 (observed); $[M+Na]^+$ = 424.50 (calculated); 424.15 (observed) (SchemeS5).

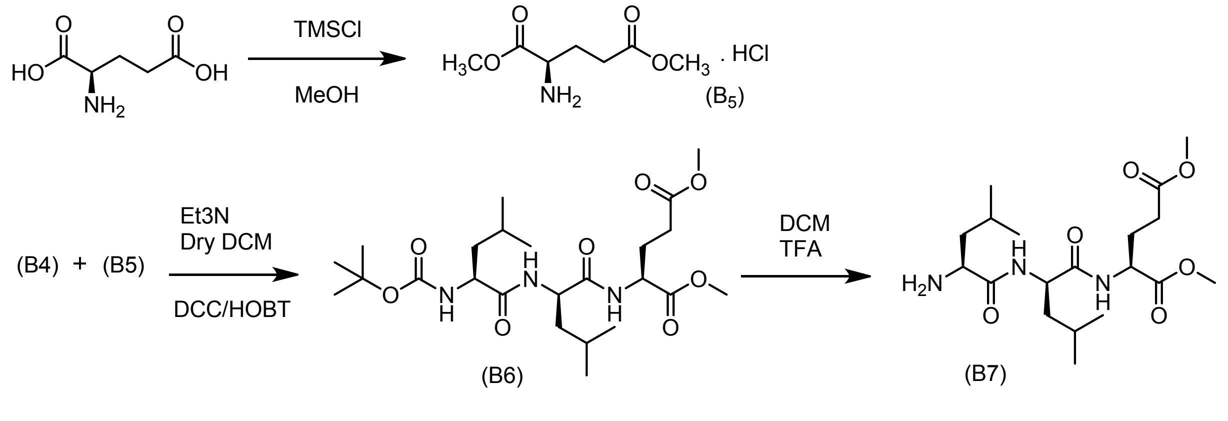

Scheme S5: Synthetic methodologies adopted for the synthesis of **B5, B6** and **B7**.

Synthesis of PFB-NH-Leu-Leu-Glu-(OMe)$_2$ (B8): 1.0 gm (2.49 mmol) of NH_2-Leu-Leu-Glu-(OMe)$_2$ was dissolved in 40 ml of methanol. Then 2,3,4,5,6-Pentafluoro benzaldehyde (0.54g, 2.73mmol) added to the methanolic solution of B7. The resulting mixture was stirred for 8h. After the reaction was completed (confirmed by TLC) yellow solid precipitate appeared. This precipitate was filtered off and washed with n-hexane and cold methanol and dried in desiccator to afford PFB-NH-Leu-Leu-Glu-(OMe)$_2$ (B8) as a pure product. Yield: 1.05gm (1.81 mmol, 72.91%). ^{1}H NMR ($CDCl_3$, 500MHz, δppm): 8.31-8.30(d, 1H, PFB), 7.62-7.60 (d, 1H, NH), 4.46-4.42 (q, 1H, C_αH,leu), 4.33-4.32 (d, 1H, C_αH,leu), 4.03-4.00 (t, 1H, C_αH, glu) 3.66 (s, 3H, -OMe), 3.57 (s, 3H, OMe) 3.10-3.05(q, 2H, C_βH, glu), 2.33-2.30 (t,2H, $CH_{2,}$glu) , 2.14-1.89 (m, 2H, C_βH, leu) , 1.67-1.61 (m, 2H, C_βH, leu), 1.56-1.53 (m, 2H, CH, leu), 0.86-0.78(m, 12H, CH_3, leu). ESI-MS (m/z): [M] =579.56 (calculated); 579.25 (observed) (SchemeS6).

Synthesis of PFB-NH-Leu-Leu-Glu-(OH)$_2$ (PA2): 1.0 gm (1.72 mmol) of PFB-NH-Leu-Leu-Glu-(OMe)$_2$ (B8) was dissolved 30 mL MeOH. Then 2M 10 mL NaOH was added and the progress of saponification was monitored by thin layer chromatography (TLC). The reaction mixture was stirred for 10h. After that the methanol was removed under vacuum; the remaining residue was dissolved in 40 mL of water, and washed with diethyl ether (2 X 50 mL). Then, the pH of the aqueous layer was adjusted to 2 using 1M HCl and extracted with ethyl acetate (3 X 50 mL). The organic extracts were pooled, dried over anhydrous sodium sulfate, and evaporated under vacuum to obtain the compound as a semi solid. Yield: 638 mg (1.15 mmol, 67.29%). ^{1}H NMR ($CDCl_3$, 500 MHz, δppm): 11.96 (s,2H, Asp-COOH) , 8.37 (s,1H, PFB, CH=N) 7.16 (d, *J*=9 Hz, 1H, NH), 7.67 (d, *J*=7.5 Hz, 1H, NH), 4.52-4.48 (m, 1H, C_αH, Glu), 4.41-4.37 (m, 1H, C_αH, Leu) 4.07 (d, *J*=7Hz, 1H, C_αH, Leu) 2.43-2.32 (m, 2H, CH_2-Glu), 2.21-2.14 (m, 1H, CH_2-Glu), 2.01-1.94 (m, 1H, CH_2-Glu), 1.74-1.57 (m, 6H, C_βH,CH_2-Leu, -CH-Leu) 0.92-0.84 (m,12H, CH_3), ESI-MS (m/z): $[M+Na+2H]^+$ =576.50 (calculated); 576.18 (observed) (SchemeS6). Elemental analysis calcd (%): ($C_{24}H_{30}F_5N_3O_6$): C 52.27, H 5.48, N 7.62, O 14.41; found: C 51.76, H 5.29, N 7.93.

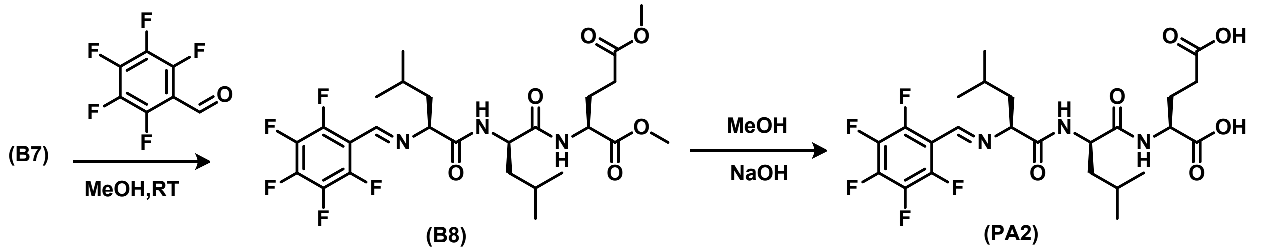

Scheme S6: Synthetic methodologies adopted for the synthesis of **B8** and **PA2**.

Synthesis of Reference compound PFB Val-Val- Glu-$(OH)_2$ (PA3)

Synthesis of BOC-NH-Val-Val-Glu-$(OMe)_2$ (C6) : 1.5g (4.74 mmol) of BOC-NH-Val-Val-OH was dissolved in 30mL dry DCM in an ice-water bath. NH_2-Leu-$(OMe)_2$.HCl 1.20 g (5.67 mmol) and Et_3N (5 ml,35.87 mmol) were then added to the reaction mixture, followed immediately by the addition of 1.16 g (5.67mmol) dicyclohexylcarbodiimide (DCC) and 0.86 g (5.67mmol) of HOBt. The reaction mixture was allowed to warm-up to RT and was stirred for 48 h. DCM was evaporated and the residue was dissolved in ethyl acetate (40 mL). The dicyclohexylurea (DCU) was filtered off. The organic layer was washed with 2M HCl (3 X 50 mL), brine (2 X50 mL), 1 M sodium carbonate (3 X 50 mL), and brine (2 X 50 mL), and finally dried over anhydrous sodium sulfate. It was then evaporated using vacuum to yield Boc-NH-Val-Val-Glu-$(OMe)_2$ as a white solid. The product was purified by silica gel (100–200 mesh) using n-hexane–ethyl acetate (3:1) as eluent. Yield: 1.98 g (4.18 mmol, 88.68%). ^{1}H NMR ($CDCl_3$, 500 MHz, δ ppm) : 7.12-7.11(d, 1H, NH), 6.74-6.72 (d, 1H, NH), 5.28-5.26 (d, 1H, NH), 4.61-4.57 (m, 1H, , $C_\alpha H$, asp),4.35-4.32(t, 1H, , $C_\alpha H$, val) 3.99-3.93 (t, 1H, $C_\alpha H$, glu) 3.74 (s, 3H, -OMe), 3.67 (s, 3H, OMe) ,2.42-2.36 (m, 2H, $C_\beta H$, val) 2.24-2.20 (m, 2H, $C_\gamma H$, glu) 2.14-1.99 (m, 2H, $C_\beta H$, glu) 1.44 (s, 9H, Boc) 0.99-0.92 (m,12H, CH_3). ESI-MS (m/z): $[M+H]^+$ = 474.27 (calculated); 474.28 (observed) (SchemeS7).

Synthesis of NH_2-Val-Val-Glu-$(OMe)_2$ (C7): 1.50 g (3.16 mmol) of Boc-NH-Val-Val-Glu-$(OMe)_2$ was dissolved in 30 mL of DCM in an ice bath. Then, 5 mL of TFA was added and stirred for 2h. The progress of the reaction was monitored by TLC. After the reaction was completed, all solvents were evaporated in a rotary evaporator. The product was then dissolved in water, neutralized with $NaHCO_3$ solution, extracted with ethyl acetate, dried over anhydrous

sodium sulfate and evaporated by rotary evaporator to obtain an oily product, which was immediately used for the next reaction. Yield: 1.10 g (2.94 mmol, 94.01%) (SchemeS7).

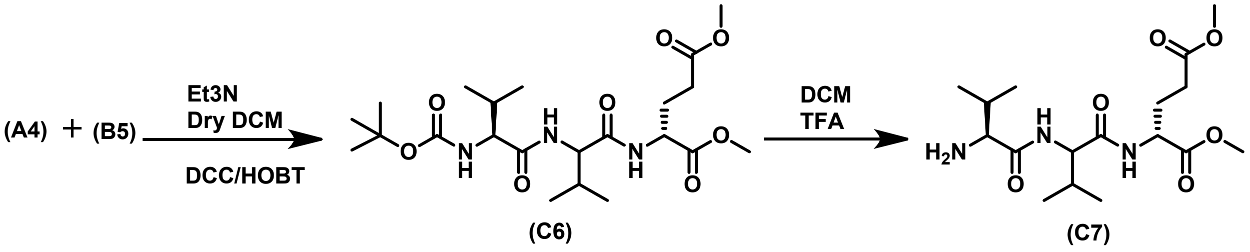

Scheme S7: Synthetic methodologies adopted for the synthesis of **C6** and **C7.**

Synthesis of PFB-NH-Val-Val-Glu-(OMe)$_2$ (C8): 1.0 g (2.68 mmol) Of NH_2-Val-Val-Glu-(OMe)$_2$ was dissolved in 40 ml of methanol. Then 2,3,4,5,6-Pentafluoro benzaldehyde (0.57g, 2.94mmol) added to the methanolic solution of **C7**. The resulting mixture was stirred for 8h. After the reaction was completed (confirmed by TLC) white solid precipitate appeared. This precipitate was filtered off and washed with n-hexane and cold methanol and dried in desiccator to afford PFB-NH-Val-Val-Glu-(OMe)$_2$ (**C8**) as a pure product. Yield: 1.12g (2.03 mmol, 76.19%). ^{1}H NMR ($CDCl_3$, 500MHz, δppm): 8.30 (s,1H, PFB, CH=N) 7.24-7.22 (d, 1H, NH), 6.91-6.89 (d, 1H, NH), 4.55-4.51 (dd, 1H, C_αH, glu), 4.37-4.33 (m, 1H, C_αH, val), 4.24-4.16 (m, 1H, C_αH, val) , 3.72 (s, 3H, OMe), 3.64 (s, 3H, OMe), 2.45-2.31 (m, 2H, C_βH, glu), 2.24-2.20 (m, 1H, C_βH, val) 2.01-1.92 (m, 2H, C_γH, glu) , 1.26 (broad peak, 1H, C_βH, val), 1.03-0.94 (m, 12H, CH_3). ESI-MS (m/z): $[M+H]^+$ = 552.20 (calculated); 552.21 (observed) (SchemeS8).

Synthesis of PFB-NH-Val-Val-Glu-(OH)$_2$ (PA3): 1g (1.81 mmol) of PFB-NH-Val-Val-Asp-(OMe)$_2$ (C8) was dissolved in 30 mL MeOH. Then 2M 5 mL NaOH was added and the progress of saponification was monitored by thin layer chromatography (TLC). The reaction mixture was stirred for 10h, the methanol was removed under vacuum; the residue was dissolved in 50 mL of water, and washed with diethyl ether (2 X 50 mL). Then, the pH of the aqueous layer was adjusted to 2 using 1M HCl and extracted with ethyl acetate (3 X 50 mL). The collected organic layer pooled and dried over anhydrous sodium sulfate, and evaporated under vacuum to obtain the desired di-carboxy compound as a semi solid. Yield: 656 mg (1.25 mmol, 69.2%). ^{1}H NMR ($CDCl_3$, 500MHz, δppm): 10.4 (s,2H, Glu-COOH), 8.32 (s,1H, PFB, CH=N) 7.26-7.25 (d, 1H,

NH), 6.93-6.91 (d, 1H, NH), 4.58-4.54 (dd, 1H, $C_{\alpha}H$, glu), 4.40-4.37(m, 1H, $C_{\alpha}H$, val), 4.21-4.18 (m, 1H, $C_{\alpha}H$, val), 2.44-2.33 (m, 2H, $C_{\beta}H$, glu), 2.27-2.22 (m, 1H, $C_{\beta}H$, val), 2.06-1.94 (m, 2H, $C_{\gamma}H$, glu) , 1.27 (broad signal, 1H, $C_{\beta}H$, val), 1.05-0.96 (m, 12H, CH_3). ESI-MS (m/z): $[M]^+$ = 523.17 (calculated); 523.30 (observed) (SchemeS8).

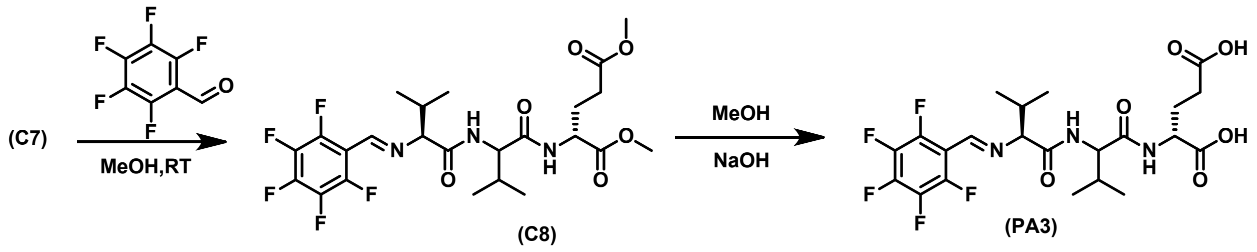

Scheme S8: Synthetic methodologies adopted for the synthesis of **C8** and **PA3**.

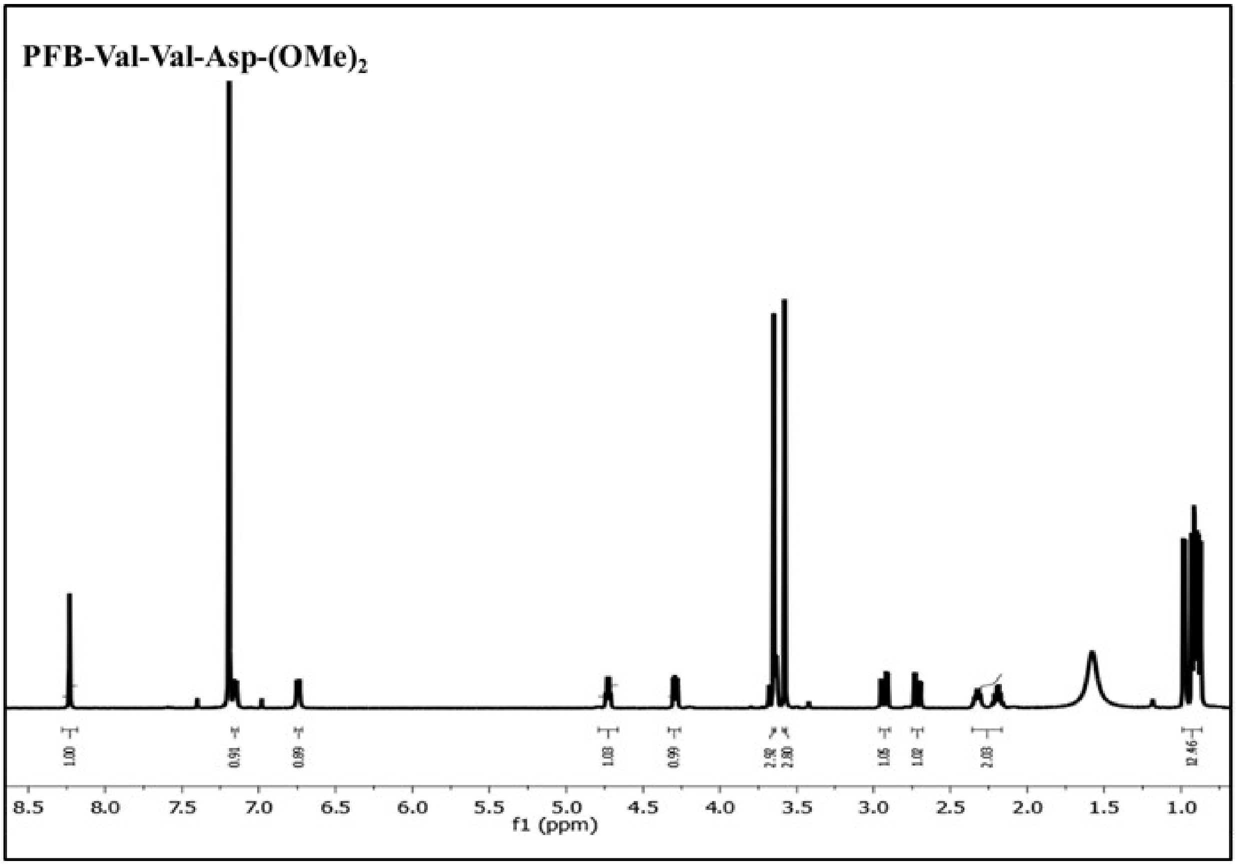

Figure S1. ^{1}H NMR ($CDCl_3$, 500 MHz, δppm) of PFB-Val-Val-Asp-$(OMe)_2$ (**A8**).

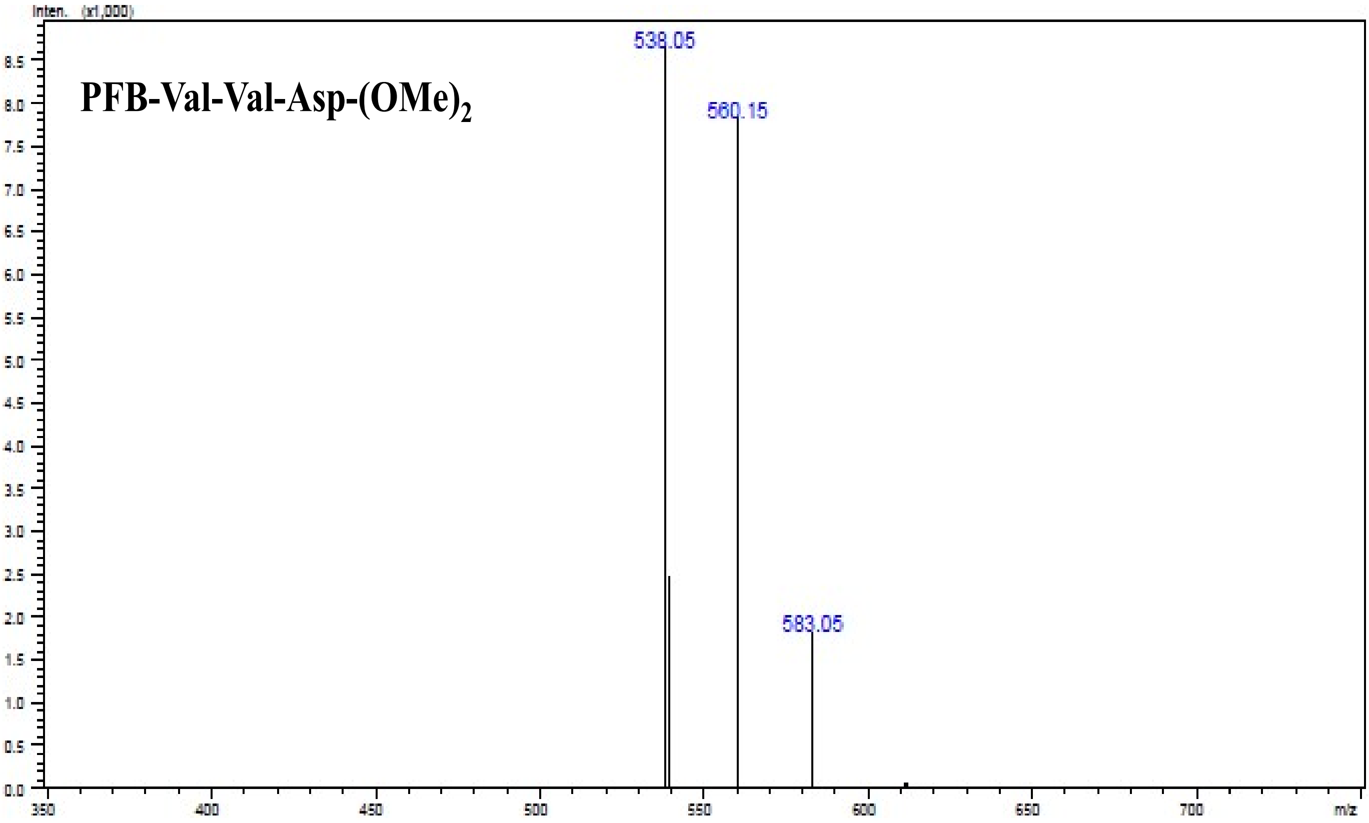

Figure S2. ESI Mass spectra of PFB-Val-Val-Asp-$(OMe)_2$ (**A8**).

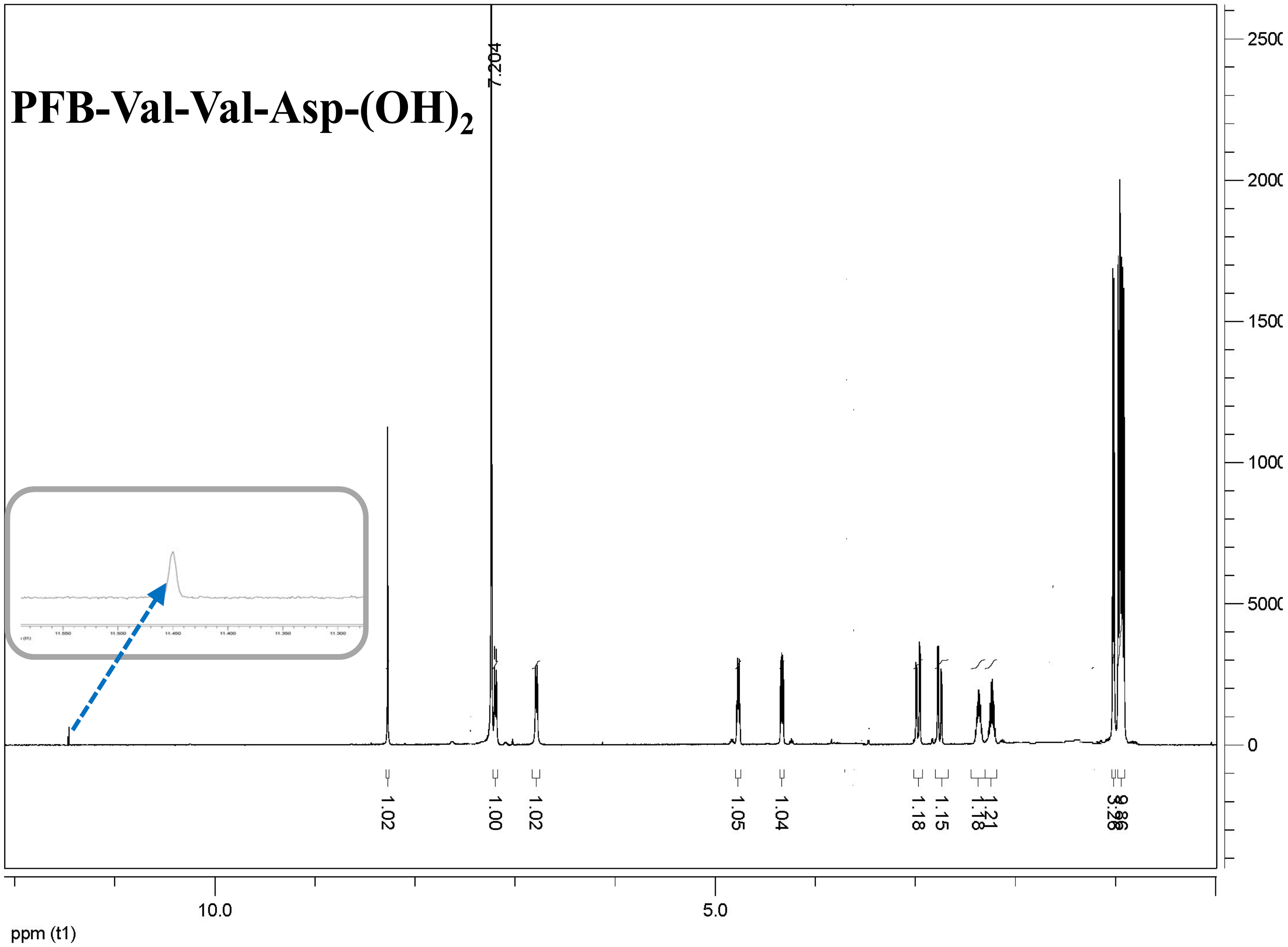

Figure S3. ^{1}H NMR ($CDCl_3$, 500 MHz, δppm) of PFB- Val-Val-Asp-$(OH)_2$ (**PA1**).

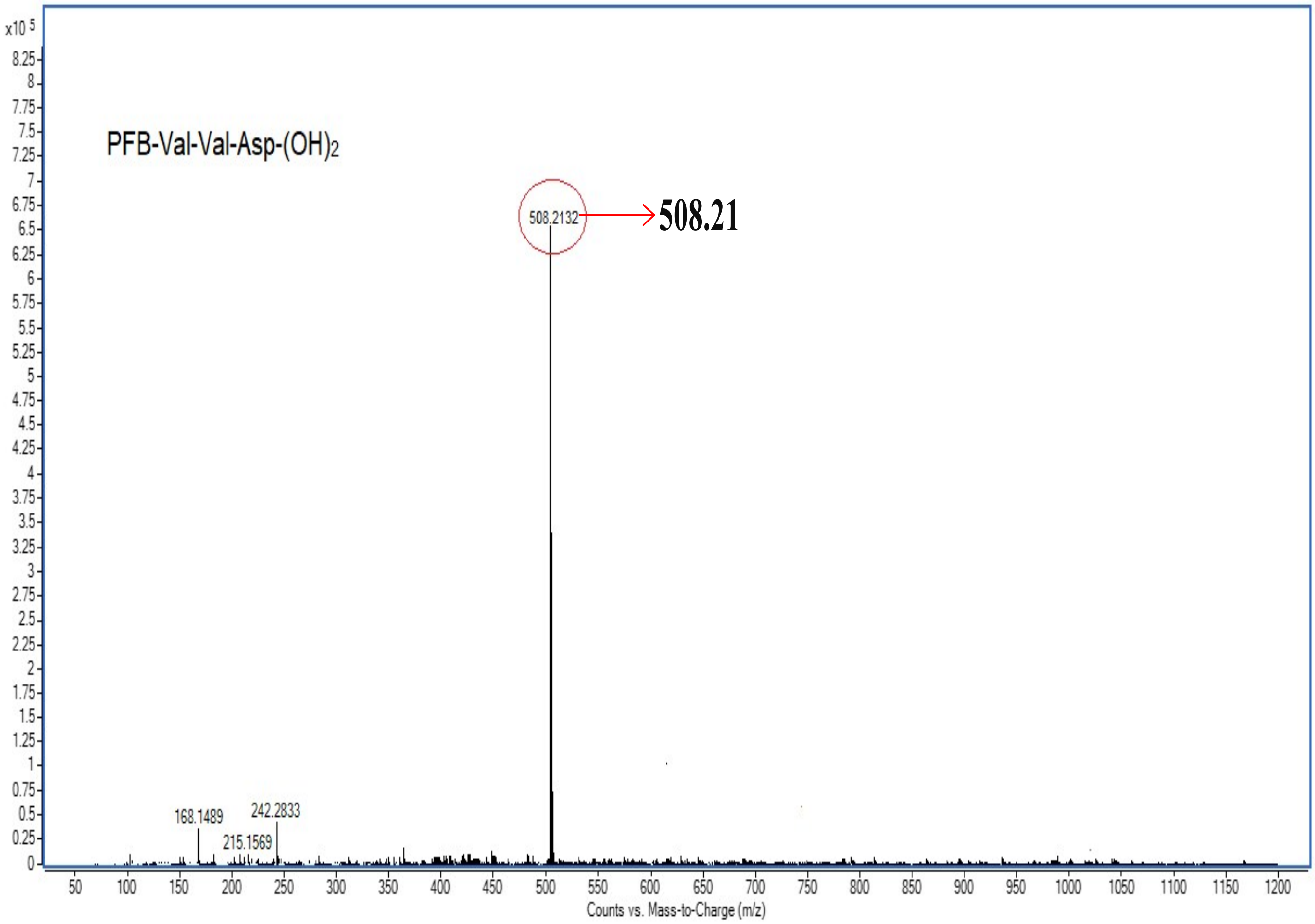

Figure S4. ESI Mass spectra of PFB- Val-Val-Asp-$(OH)_2$ (**PA1**) .

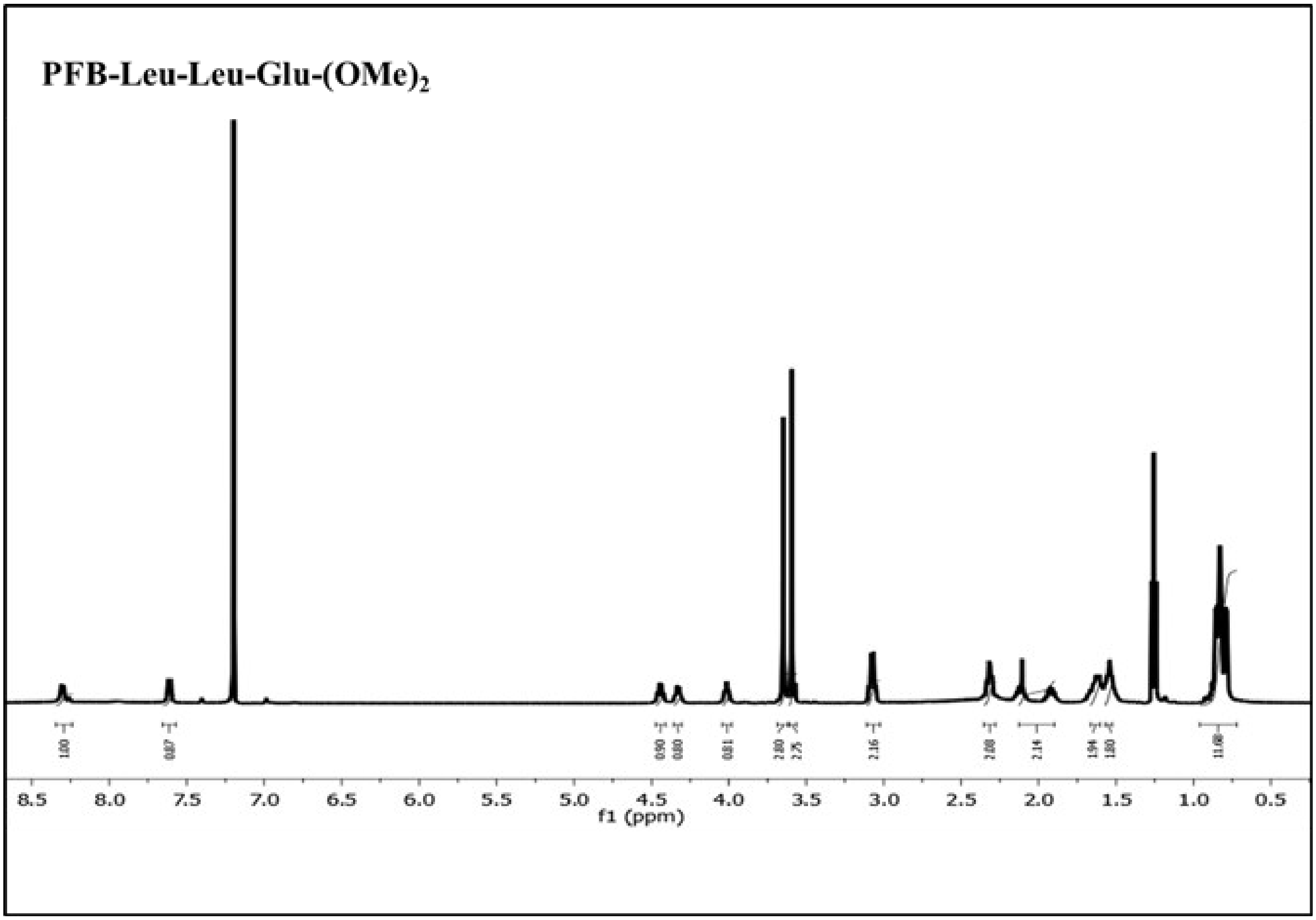

Figure S5. ^{1}H NMR ($CDCl_3$, 500 MHz, δppm) of PFB- Leu-Leu-Glu-(OMe)$_2$ (**B8**).

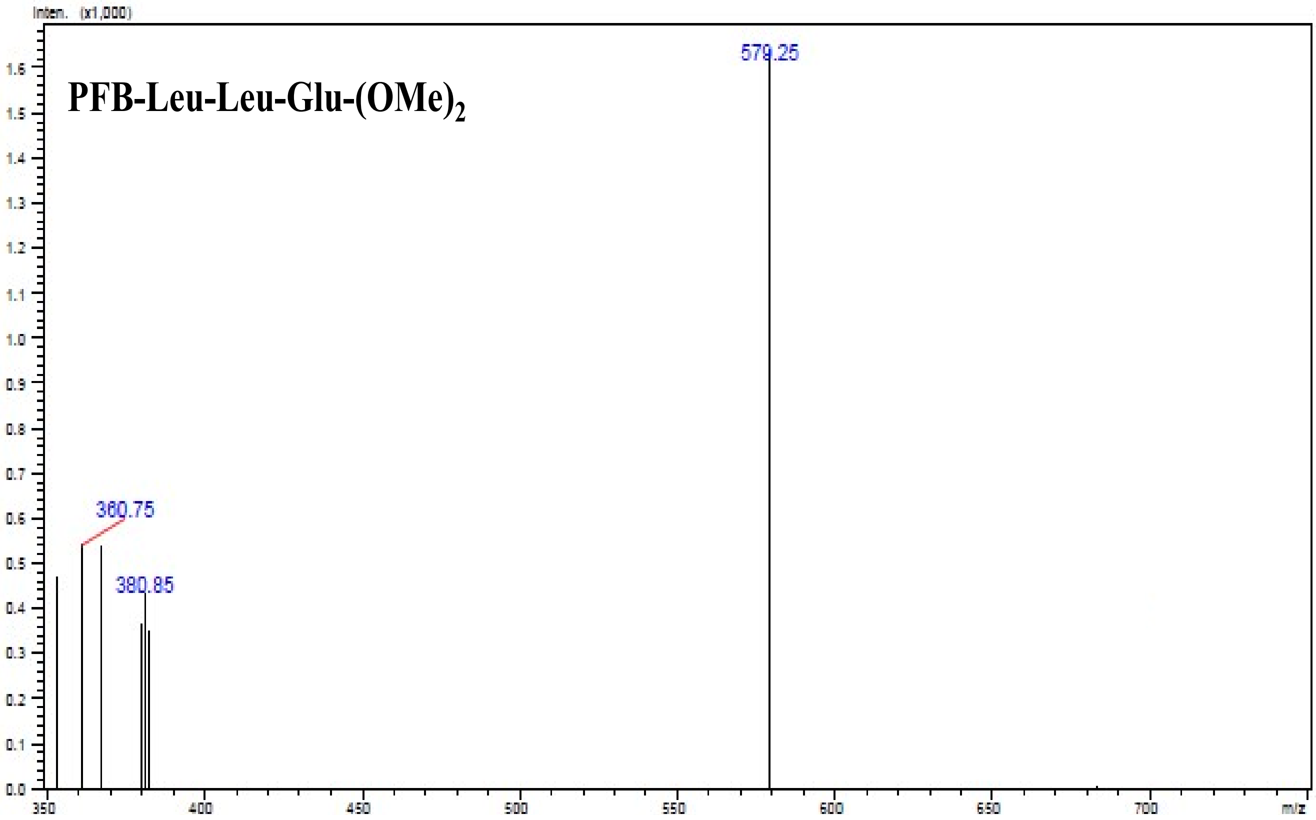

Figure S6. ESI Mass spectra of PFB-Leu-Leu-Glu-(OMe)$_2$ (**B8**).

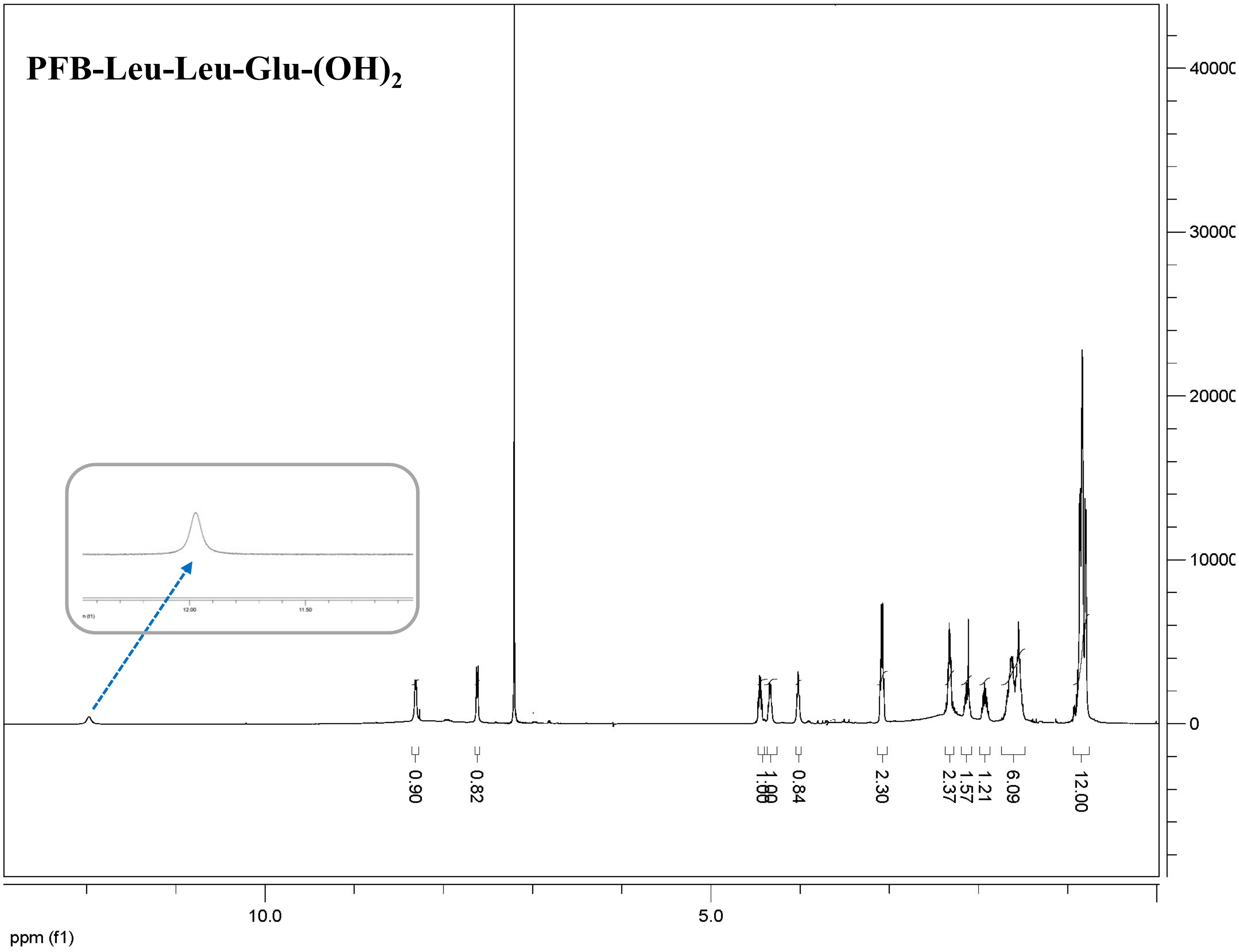

Figure S7. ^{1}H NMR ($CDCl_3$, 500 MHz, δppm) of PFB- Leu-Leu-Glu-(OH)$_2$ (**PA2**).

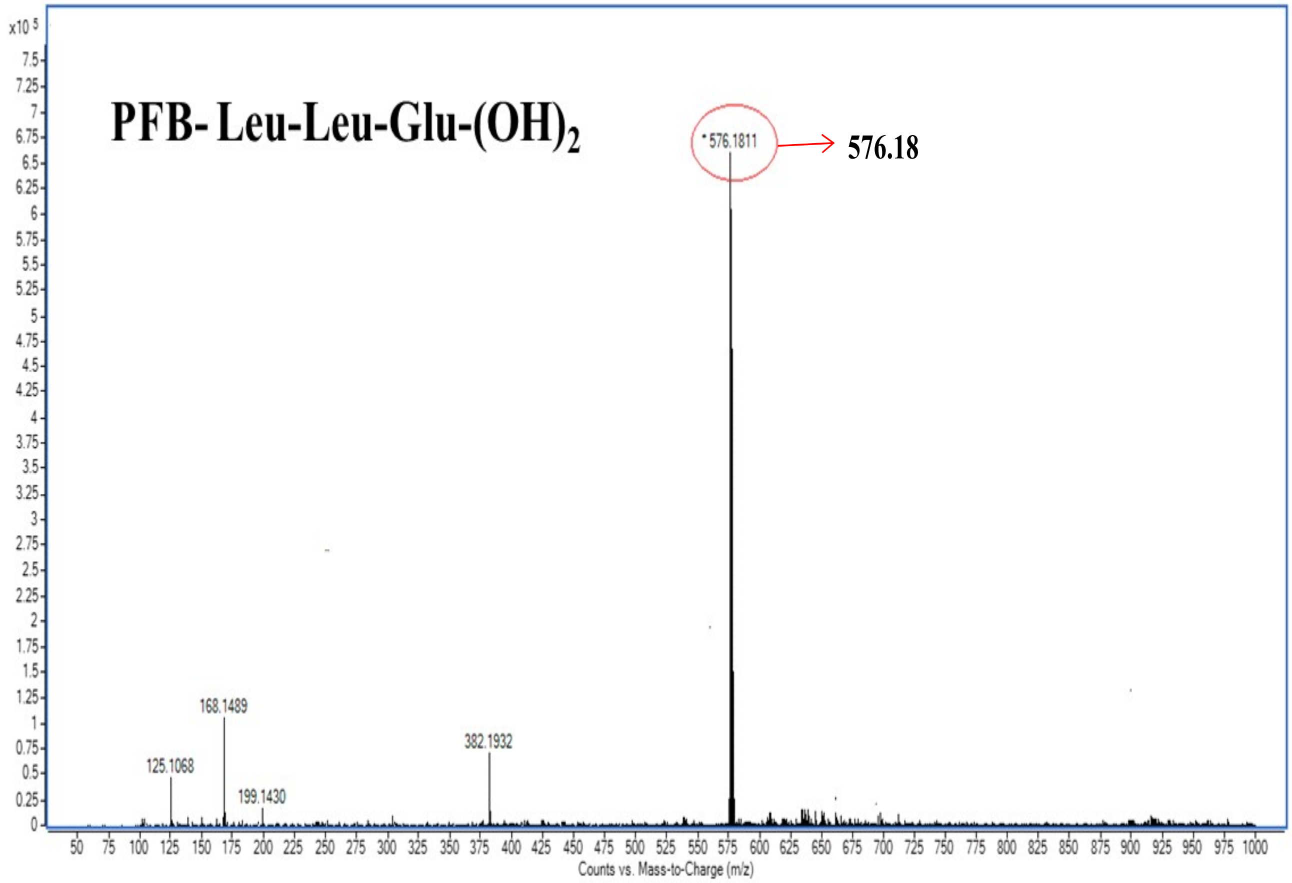

Figure S8. ESI Mass spectra of PFB-Leu-Leu-Glu-$(OH)_2$ (**PA2**).

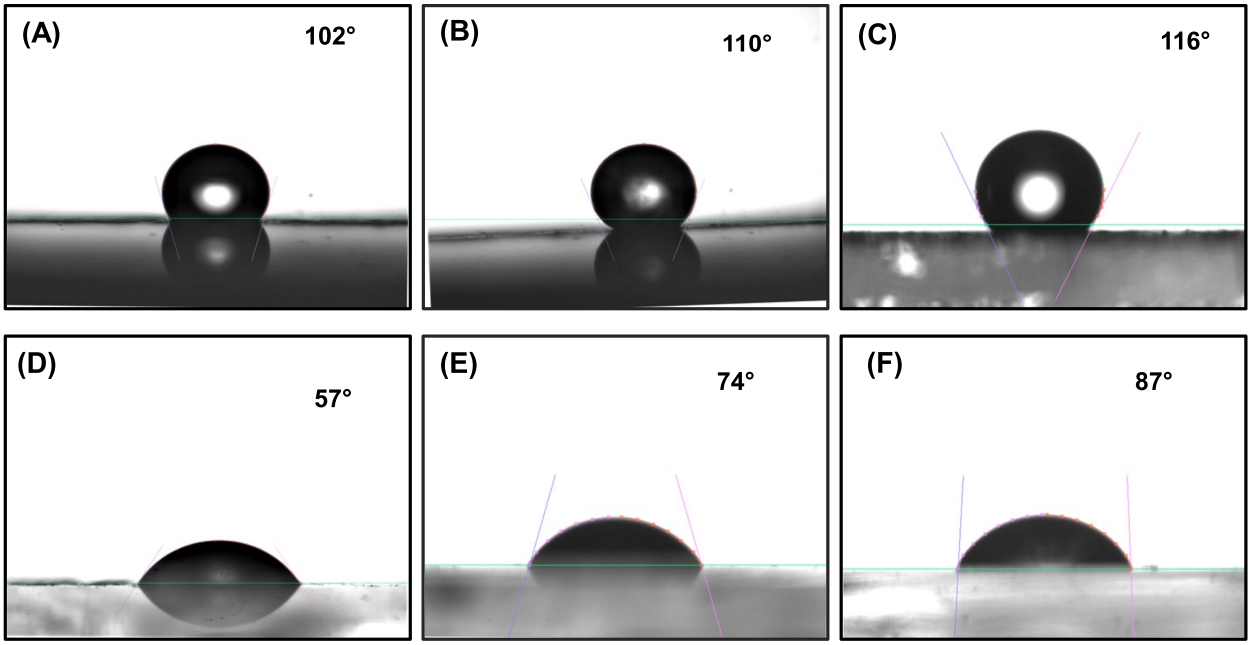

Figure S9. Contact angle measurements of silica surface coated with **PA1** at different concentrations: (A) 1mg/mL, (B) 3mg/mL, (C) 5mg/mL and with **PA2** at different concentrations: (D) 1mg/mL, (E) 3mg/mL, (F) 5mg/mL.

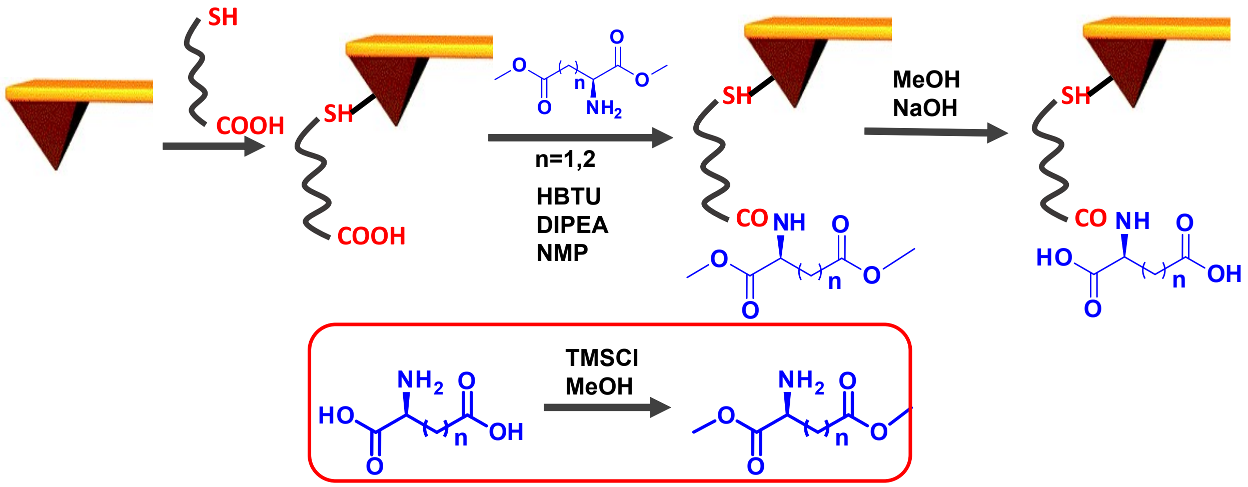

Figure S10. Schematic representation of the chemical modification procedure of the gold AFM tip required for force measurement study.

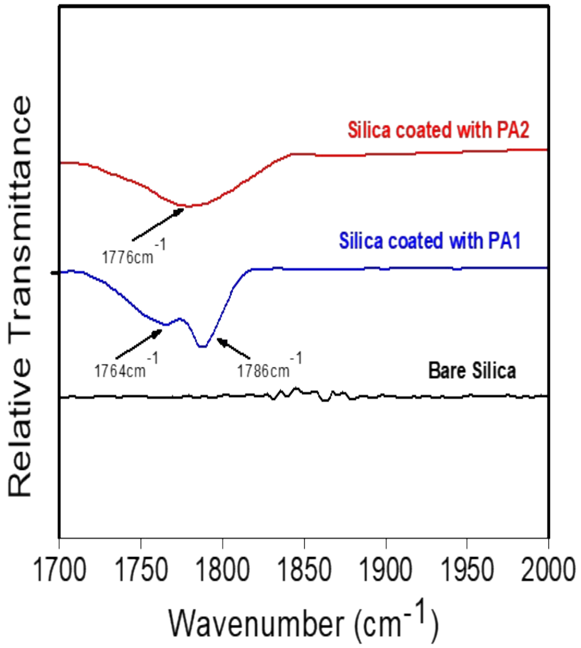

Figure S11. ATR-FTIR spectra of bare silica surface (black) and coated with peptides **PA1** (blue) and **PA2** (red).

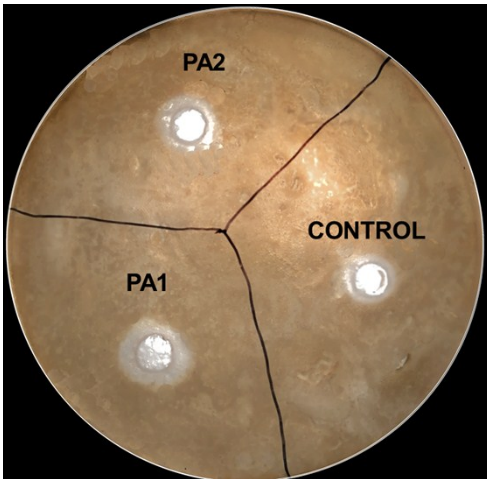

Figure S12. Representative image of the zone of inhibition obtained for *E.coli* with or without the compounds.

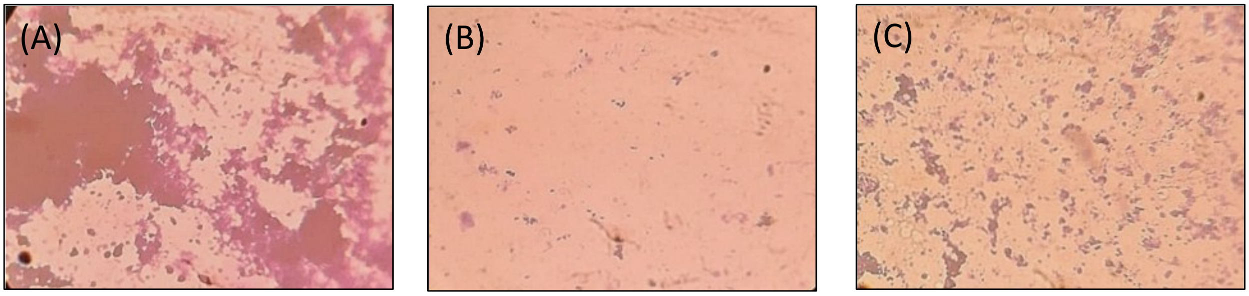

Figure S13. Representative light microscopy images of bacterial adhesion (*Pseudomonas aeruginosa*) on surface either uncoated (A) or coated with peptides **PA1** (B) and **PA2** (C)

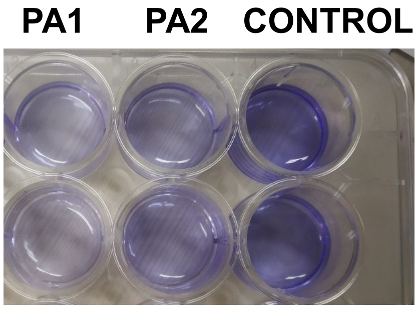

Figure S14. Representative image of the Crystal violet assay after addition of acetic acid (before spectrophotometric analysis) obtained for *E.coli* with or without the peptides (**PA1** and **PA2**).

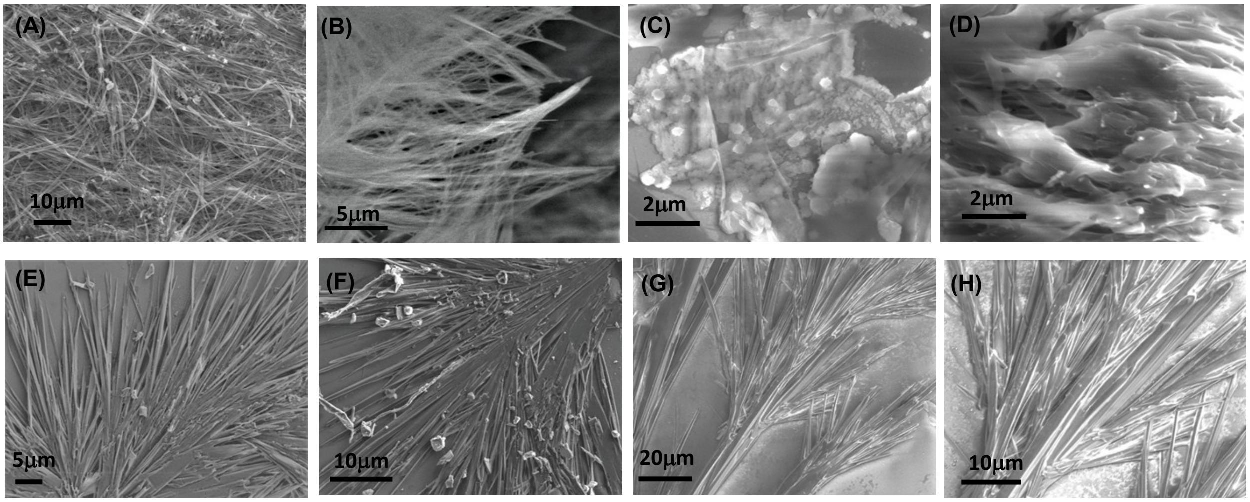

Figure S15. FE-SEM micrographs of the self-assembled structure formed by **PA1** in 100% ethanol (A, B), in 100% water (C, D) and **PA2** in 100% ethanol (E, F), in 100% water (G, H).

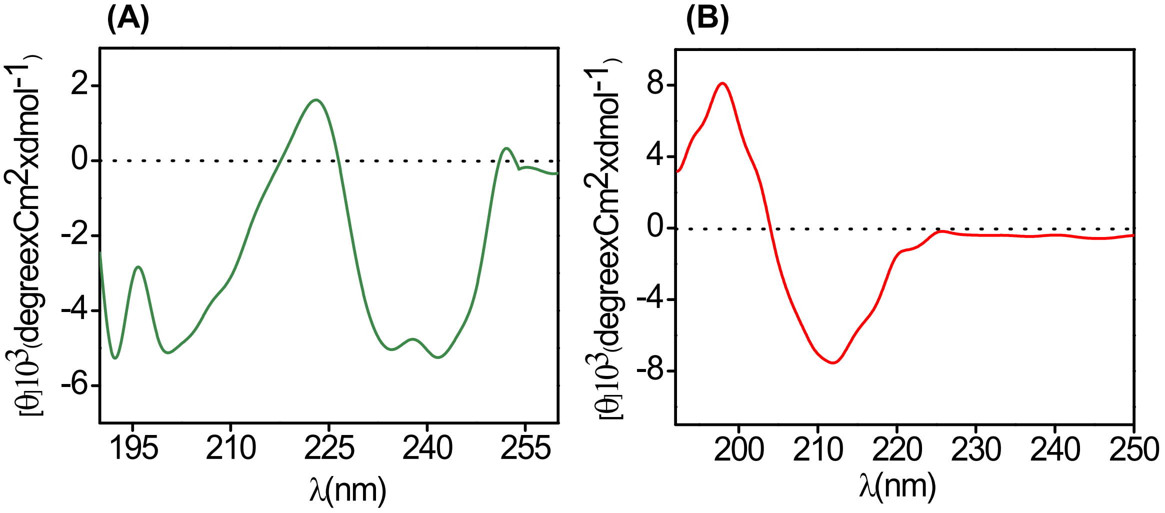

Figure S16. Circular dichroism (CD) spectra of **PA1** (A) and **PA2** (B).

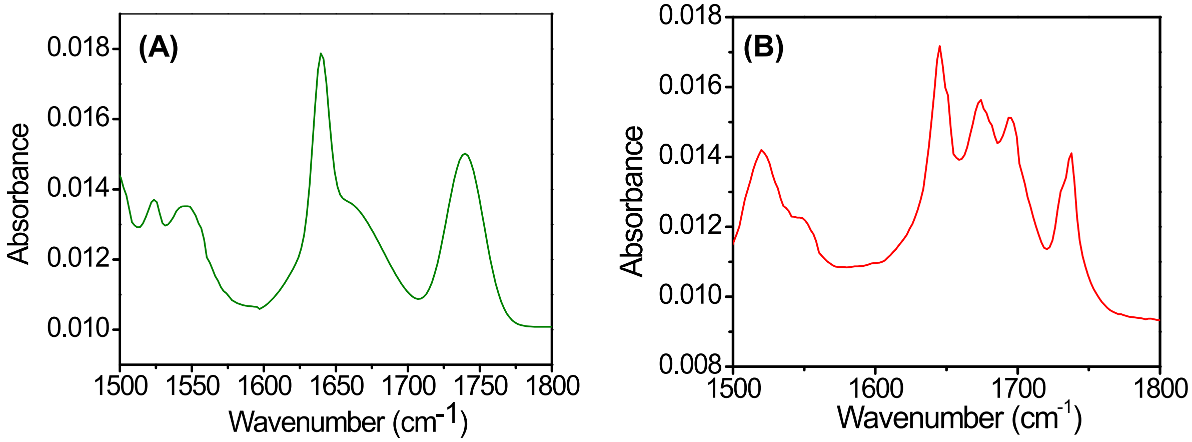

Figure S17. FT-IR spectra of the self-assembled structures formed by **PA1** (A) and **PA2** (B) having both amide I and II region.

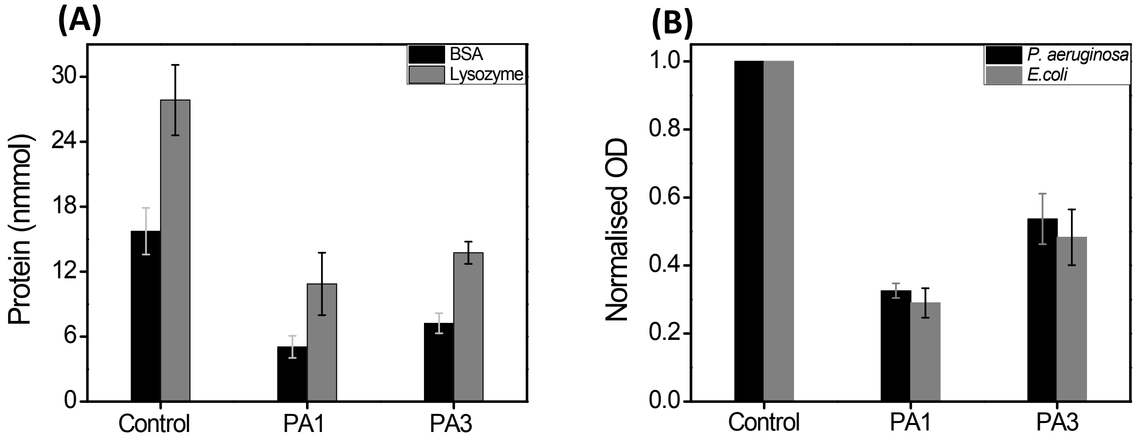

Figure S18. (A) Adsorbed amounts of BSA (black) and lysozyme (grey) to bare (control) and coated silica surface with peptides **PA1** and **PA3**. (B) Normalized optical density quantification of the accumulated P. aeruginosa (black) and E.coli (grey) on bare (control), **PA1** and **PA3** coated silica surface.

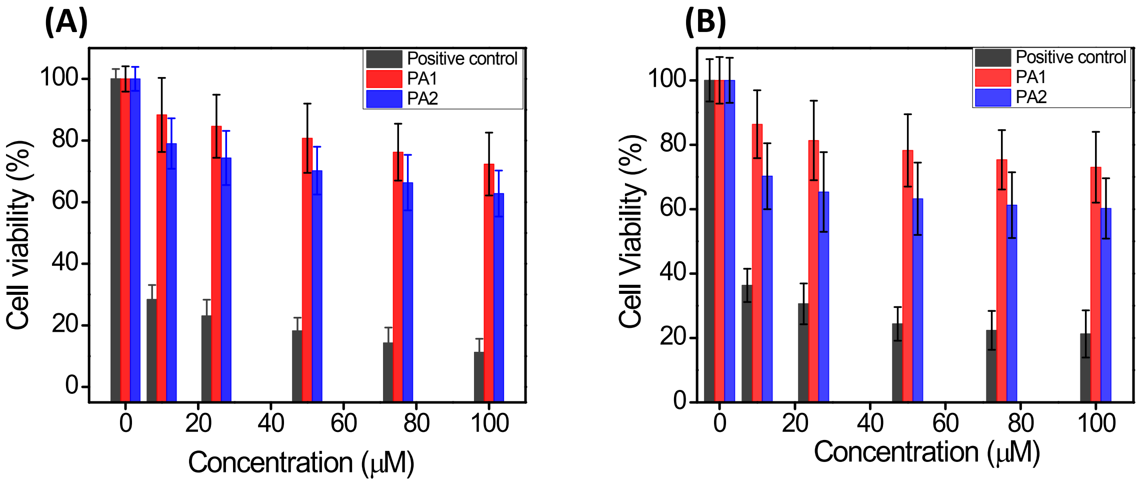

Figure S19. (A) Cell viability values (%) estimated by MTT cell viability test for both **PA1** and **PA2**. HEK 293 cells were cultured in the presence of the peptides at 37°C for 24 h (% viability was calculated considering 100% growth in the absence of peptides); (B) Percentage cell viability in HEK 293 cells as determined by Trypan blue after 24h incubation of peptides **PA1** or **PA2**. (% viability was calculated considering 100% growth in the absence of peptides). The error bars represent standard deviation (n=3).
